# Constructing an evaluation model for the comprehensive level of sustainable development of provincial competitive sports in China based on DPSIR and MCDM

**DOI:** 10.1371/journal.pone.0301411

**Published:** 2024-04-16

**Authors:** Ke Xu, Hung‐Lung Lin, Jianna Qiu

**Affiliations:** 1 School of Physical Education, Shanghai University of Sport, Shanghai, China; 2 College of Physical Education, Quanzhou Normal University, Quanzhou, China; 3 School of Economics and Management, Sanming University, Sanming, China; 4 School of Foreign Languages, Quanzhou Normal University, Quanzhou, China; Gonbad Kavous University, IRAN, ISLAMIC REPUBLIC OF

## Abstract

This study focuses on the objective assessment of sport development in socio-economic environments, considering the challenges faced by the industry. These challenges include disparities in regional investments, limited market participation, slow progress towards sports professionalization, and insufficient technological innovations. To tackle these challenges, we suggest implementing an integrated evaluation model that follows the DPSIR (Drivers, Pressures, States, Impacts, Responses) framework and incorporates comprehensive socioeconomic indicators. Subsequently, we utilized the Entropy power method and TOPSIS (Order Preference Technique for Similarity to an Ideal Solution, TOPSIS) analysis to comprehensively assess the progress of competitive sports development in 31 provinces and cities in China. Additionally, we recommended further developments in competitive sports and proposed precise strategies for promoting its growth. The framework and methodology developed in this paper provide an objective and scientifically based set of decision-making guidelines that can be adopted by government agencies and related industries in order to create successful plans that promote the sustainable growth of competitive sport. This is expected to bolster the nation’s global influence, enhance social unity, and fuel economic expansion. The findings of this study offer policymakers valuable insights regarding competitive sports and can advance the development of the sports sector in China, thus making it a crucial driver of regional socio-economic progress.

## Introduction

### Background and motivation of the study

With the progress of human civilization, the continuous development of social economy, culture and science and technology, and the accompanying drastic changes in the way of life of human beings, competitive sports have become more and more a symbol of national soft power. It plays an irreplaceable role in uplifting the national spirit and enhancing national cohesion. The primary purpose of competitive sports in China was initially to secure medals in international competitions. However, with the changes in society, competitive sports have not only enhanced the country’s soft power but also assumed an important role in promoting economic development. In particular, competitive sports play a significant role in promoting the development of the domestic economy and building new economic growth points. The interaction between competitive sports and the social economy has become increasingly evident. Sport is an important part of socio-economic development, and competitive sport is a major contributor to regional economic development.

According to data from the General Administration of Sport of China and the National Bureau of Statistics, China’s competitive sports have achieved a total of 251 medals at the London, Rio, and Tokyo Olympic Games from 2013 to 2022, thanks to consistent investment. Of these, 967 world championships and 113 world records were won in various international competitions, including the Olympic Games. The development of the sports industry has been greatly boosted by the long-standing high-level competitive sports, resulting in the total size of China’s sports industry increasing from 1.1 trillion yuan to 3.5 trillion yuan between 2013 and 2022. The investment has increased by 2.4 trillion yuan over the course of ten years. The sports industry’s share of GDP rose from 0.63% to 2.89%, representing a 459% increase over a span of 10 years. It can be seen that competitive sports not only reflect the soft power of a country but also play an important role in promoting social and economic development. Hence, the sustainability of competitive sports programs is an important research issue. The boost to domestic consumption from competitive sporting events is also significant. According to the Guangzhou Sports Bureau and the Guangzhou Bureau of Statistics, taking the 2010 Guangzhou Asian Games as an example, not only did China rank first in the medal table with 199 gold medals, 119 silver medals, and 98 bronze medals, but also the “Asian Games effect” brought direct economic benefits of up to 800 billion yuan to the Guangzhou. In 2010, Guangdong’s GDP surpassed one trillion yuan and Guangzhou’s total tourism revenue exceeded 100 billion yuan, reaching 125.461 billion yuan, representing a year-on-year increase of 26.21%. It also promoted the construction of stadiums and the rapid development of the sports industry. In 2013, the number of stadiums in Guangzhou doubled compared to 2003, and the added value of the sports industry was 28.22 billion yuan [1] accounting for 1.84% of the city’s GDP, which maintained a high rate of growth for six consecutive years. The importance that the Chinese government attaches to competitive sports is reflected in the documents it has issued.

Since 1984, when China won its first Olympic gold medal, the General Administration of Sport (GAS) and the State Council have introduced many policies to promote the quality and sustainable development of competitive sports, and through these policies, the country’s soft power is enhanced to promote China’s social and economic development. For example, Beijing’s GDP increased by 105.5 billion yuan from 2004 to 2008 as a result of hosting the 2008 Olympic Games, according to the National Bureau of Statistics. The average annual GDP growth was 12.3% from 2005 to 2007, and 1.82 million job opportunities were created in the Beijing area. The “Olympic effect” has also contributed to the long-term development of the Beijing area, with an average annual growth rate of 5% for domestic tourists and 8% for foreign tourists. Additionally, the city received 4 million domestic and foreign tourists during the Beijing Olympics. In the period from 2001 to 2007, Beijing experienced an average annual increase of over RMB 15 billion in retail sales of consumer goods, attributed to the Olympic Games. This resulted in a total increase of RMB 110 billion in retail sales of consumer goods over the course of seven years. From the 2008 Beijing Olympics to the 2022 Beijing Winter Olympics, the Chinese government has achieved some success in international soft power and socio-economic development as a result of its active investment in the sustainable development of sport.

However, there are still many problems. These problems, which are illustrated as follows:

#### (1) The problem of unbalanced inter-regional development

Regarding the uneven development of competitive sports, Ma and Kurscheidt’s [2] argued that the allocation of resources to competitive sports in China is measured by the number of medals won by athletes affiliated with provincial sports bureaus (PSBs) at the National Games of China (NGC). The provincial government (PG) provides incentives to its athletes and coaches based on the number of medals won at the NGC, and the salaries of PSB managers at all levels are paid by the PG rather than the General Administration of Sport (GAS). The only criteria for promotions or salary increases for PSB personnel at all levels is also based on the number of medals won at the NGC by athletes from the provinces to which the PSB belongs. The number of medals won in the NGC becomes a clear indicator of the performance of competitive sports in a province. The PSB’s excessive focus on the number of NGC medals inevitably leads to a rush for high-level athletes in the transfer market for elite athletes, and economic strength becomes the most critical indicator of whether a province can obtain high-level athletes. This has led to an imbalance in the distribution of competitive sports resources due to the disparity in regional economic strength, with some regions becoming the gathering place for elite athletes due to the development of competitive sports, while other economically backward regions have few people seeking them. This leads to a significant Matthew effect in provincial sports, and in the long run, the sustainable development of competitive sports in China will be out of reach.

#### (2) Insufficient market participation

Yang [3] stated, as observed from the effects of the Chinese government’s long-term policy of promoting competitive sports, the government’s investment of a large amount of human, material and financial resources in the short and medium term (5–15 years) can yield significant benefits for the country’s economic and social development. However, after the event, the large sports venues or large hotels may be unused and rarely used. In the long run, there is no effective synergy between the sports industry and various other industries. Due to the industry’s high degree of dependence on the government, the refusal of the industry to commit resources, the dysfunctional market mechanism among various industries, and the lack of motivation to actively innovate in research and development, the sustainable development of the competitive sports industry will ultimately be significantly affected.

#### (3) The lagging behind of professionalization reforms

Peng [4] claimed that the professionalization reform is a top priority for the sustainable development of competitive sports. The government’s massive investment of resources in athletics is unsustainable, and China must reduce the amount of state funding per gold medal. Making professional clubs one of the training paths for China’s elite athletes and high-level coaches can lower the dependence of athletes and coaches on the government. In this context, the reform of the professionalization of competitive sports is of the utmost importance. Whether it is the NBA, MFL, English Premier League, Serie A, these professional sports leagues do not need the financial investment of the country in the training of world-class athletes and coaches, but also drive the development of the country’s sports industry, boosting employment, and creating extremely high economic value. Thus, a world-class professional league is the key to the sustainable and high-quality development of competitive sports in China in the future. In this regard, China’s professionalization reform of competitive sports is still lagging a bit behind and needs to catch up.

#### (4) Low level of technology empowerment

Science and technology will undoubtedly play a pivotal role in the future of global competition in sports. The rise of competitive sports in the UK in the last decade or so reflects this trend effectively. The lack of scientific and technological advancement in China’s competitive sports is evident in the insufficient scientific level of sports training, the limited capacity of scientific training In the new stage of development, science, and technology are important for enhancing the efficiency of competitive sports and facilitating their high-quality and sustainable development in China.

#### (5) Some conflict exists between national and provincial sports organizations

Zheng et al. [5] revealed the characteristics of conflict between national and provincial sports organizations through a study of inter-organizational conflict in three sports: artistic gymnastics, swimming, and cycling, in terms of evidence of conflict, factors leading to conflict, initiatives to mitigate conflict, and the impact of conflict on elite sport. Despite the differences in the degree and characteristics of national and provincial conflicts between different sports, the causes of conflicts and measures to deal with conflicts are somewhat consistent, whether it is “horizontal coordination at the national level” or “vertical coordination between the national policy level and the region” is conducive to alleviating conflicts to a certain extent, and the effective adoption of effective measures to alleviate organizational conflicts is of great significance to improving the overall performance of China’s elite sports in the future.

#### (6) Weak succession of human resources related to competitive sports

Athletes and coaches are key to the success of competitive sports in the international arena, but as a result of China’s 30-year one-child policy and the dwindling number of elite coaches in China, the number of athletes and coaches in the country is decreasing. In the increasingly fierce international competition in competitive sports, the shortage of human resources related to competitive sports becomes a problem that should be solved in order to construct a strong sports nation. Li et al. [6] concluded that China’s one-child policy has led to a decline in the birth rate and the number of youth football participants, which would affect the development of professional football in China. Chen & Chen [7] suggested that the traditional way of training elite coaches is no longer suitable for the current competitive landscape of international sports, and that the training path of elite coaches needs to be further reformed, especially in terms of sustainable development, professional training, quality training, and the establishment of a “dual-track” training model for sports coaches. (Note: the education system and the sports system share the resources of elite coaches).

Scholars have conducted a variety of studies to address the problems of regional imbalance, insufficient market participation, low level of scientific and technological empowerment, organizational conflicts, and human resources for competitive sports, but to date, there have been no in-depth studies on the sustainable development of provincial elite sports in the Chinese context.

### Objectives of the study

In view of the above challenges, this study aims to achieve the following objectives.

(1) Integrating assessment model: An assessment model based on social and economic indicators was created to distinguish it from previous studies. The model follows the DPSIR framework and incorporates not only economic indicators, but also a range of sport- and socially-related environment indicators. By objectively evaluating social and economic factors, this study aims to assess the level of development in competitive sports across various provinces. Emphasis is placed on the interdependent relationship between sports and the economy
(2) Addressing Regional Disparities: This study aims to address the regional imbalances in the development of competitive sports. It will provide insights and recommendations to reduce the development gap between states, thus promoting more equitable growth of the national competitive sports sector.
(3) Promoting Market Participation: Addressing the challenge of insufficient market participation in competitive sport. This paper aimed to analyze the impact of long-term government investments and policies on competitive sport, and to provide recommendations for improving synergies between the sport industry and other sectors. This entails ensuring the efficient utilization of sports facilities and infrastructure beyond particular events, which can contribute to sustainable development.
(4) Facilitating Professionalization Reforms: The responsible parties should assess the advancement of professionalization reforms in competitive sports and highlight the necessity for China to reduce its reliance on extensive state funding for each gold medal. The study aims to explore the potential advantages of cultivating professional sports leagues similar to those of international competitors like the NBA, MFL, English Premier League, and Serie A. These leagues operate with limited government funding and make significant contributions to the economic and employment growth of their respective nations.
(5) Promoting Technology Enablement: The significance of science and technology in the advancement of competitive sports is highlighted. This involves improving the scientific elements of sports training, strengthening logistical support for scientific training, and integrating state-of-the-art technology into training programs. The aim is to enhance the efficiency, quality and sustainability of competitive sports through technological advancements.

### Research innovations and contributions

This study presents a groundbreaking method by creating an integrated assessment model utilizing the DPSIR framework that surpasses typical economic indicators and incorporates sport-related and socio-environmental indicators. This pioneering model offers a thorough comprehension of the correlation between competitive sport and economic progress. Moreover, the combination of entropy and Technique for Order Preference by Similarity to Ideal Solution (TOPSIS) in this study can combine subjective and objective evaluation, and its evaluation principle is clear, operable, and has a wide range of application [8]. A description of these two methods is given in more detail in research methods. Importantly, the combination of the qualitative DPSIR with the quantitative Entropy and TOPSIS models provides a valuable tool for government officials, industry leaders and policy makers. This tool aims to better guide the integration of sport into broader economic development strategies, raise awareness of the economic value of sport, and improve the planning and management of China’s sports industry.

## The DPSIR model and sustainability in sport

### The DPSIR model

Driving forces—Pressure—State—Impact—Response” is referred to as the DPSIR model. The DPSIR model was first proposed by the Organization for Economic Cooperation and Development (OECD) Niemeijer and Groot [9]. The explanation of each element of the DPSIR model is as follows [10]. (1) Driving forces refer to the underlying causes and factors that drive change in a system. Driving forces are typically social, economic, and political factors. These can include population growth, technological innovations, policy changes, and resource utilization, among others. Driving forces typically have an impact on the system and initiate a series of transformations. (2) Pressure refers to the forces or impacts exerted on a system, either from within or from external sources. These pressures can include overexploitation of resources, environmental pollution, and ecosystem destruction. Pressure usually has a negative impact on the state of the system. (3) State refers to a description or characterization of the current condition of the system. In the DPSIR model, the state typically encompasses a range of indicators and parameters in environmental, social, or economic terms. These state parameters can include water quality, air quality, biodiversity, population health, economic growth, and so on. (4) Impacts are the results or changes that occur as a result of the interaction of pressures and states. These impacts can be positive or negative, visible or latent, but they are usually used to gauge the extent to which a system has been affected. (5) Responses are measures taken by society or the government in response to pressures and impacts. This can include formulating policies, implementing regulations, environmental protection initiatives, social action, etc. The goal of the response is usually to reduce stress, improve conditions, minimize adverse impacts, and enhance system sustainability. Through this approach, policymakers and researchers can identify the key issues in the system and devise the appropriate responses to promote sustainable development and improve the overall system performance. The model has been widely used in the fields of the environmental management, the sustainability research, the policy making and the social science research.

Since it was first proposed, the DPSIR model has been widely used in sustainability planning assessment and research in environmental and economic development. For example,

(1) studies with natural resource environment: Malmir et al. [11] used DPSIR model to provide a solution to groundwater resource management problems in Najafabad region in central Iran, which provides a reference for managers and decision-makers. Wang and Yang. [12] employed the DPSIR model to comprehensively evaluate the sustainable development potential of China’s shale gas industry, by selecting economic, environmental, resource, and technological factors, and constructing an evaluation index system for the shale gas DPSIR framework. The evaluation results of the study concluded that the sustainable development potential of China’s shale gas industry is relatively lower, and that the sustainable development potential of the southwest region is better than that of the northwest region. Khan et al. [13] constructed a DPSIR analytical framework to evaluate the rural sustainable development efficiency (RSDE) in the Yellow River Basin of China, with the use of 30-year panel data from 1997–2017 in nine provinces. The results of the study showed that the initial RSDE, cropping structure, rate of financial autonomy and level of mechanization inhibited the increase in RSDE, whereas the level of urbanization and rural GDP per capita had a negative and non-significant effect on RSDE across the watershed. Hou et al. [14] utilized the DPSIR model to conduct a comprehensive evaluation of 13 prefecture-level cities in Jiangsu Province, China, with the aim of proposing a sustainable development response that integrates ecological integrity, ecosystem services, and human well-being as the province develops.

(2) Research with a focus on socio-economic development: Gupta et al. [15] applied the DPISR model to investigate the worldwide developments and challenges brought to light by the COVID-19 pandemic in 2019. They found that the affluent’s “status” and “impact” were prioritized over the poor, ignoring any potential “drivers” and “pressures” in favor of swift economic recovery. The “drivers” that caused the global spread of the pandemic were also overlooked. Second, the response disregarded the underlying causes and pressures in favor of a rapid economic recovery. Ultimately, the primary cause of the global outbreak was the utilization of government funding to recuperate from the pandemic. Ultimately, the primary cause of the global outbreak was the use of government funds to recover from the pandemic. After all, the “driver” of the global outbreak is the use of government funds to restore business as usual, which will lead to a vicious cycle of further ecological degradation, socio-economic inequality, and domestic abuse. An inclusive development approach based on the DPSIR model would lead to a virtuous circle by emphasizing human health, well-being and ecosystem regeneration. Liu et al. [16] proposed a Driver-Pressure-State-Impact-Response (DPSIR) framework to study the major socio-economic influences on SO_2_ emissions in Chinese cities. The study found that socio-economic factors, including urbanization, sustained economic growth, optimization of industrial structure, and an improvement in energy efficiency, played a significant role in lowering SO_2_ emissions in China. In contrast, the government’s mandated environmental monitoring and financial investments in technology were deemed ineffective in reducing SO_2_ emissions. Brunhara et al. [17] employed the DPISR model to develop an indicator system of 16 driver indicators (D), 74 pressure indicators (P), 23 state indicators (S), 35 impact indicators (I), and 38 response indicators (R) to help a cooperative of waste pickers in Brazil self-assess its social, environmental, and economic performance and to facilitate the cooperative’s proposal of highly targeted improvements. Kim et al. [18] In order to achieve effective, efficient and equitable global fire governance, the DPSIR framework is used to construct fire-related issues to establish a coherent causal path among drivers (D), pressures (P), states (S), effects (I) and responses (R). The overall decline in global burning area masks economic realities that increase the likelihood and cost of hazards caused by fires, suggesting the use of new indicators to assess and communicate the impact of global economic drivers on fire activity. Afrin and Shammi [19] utilized the DPSIR framework to examine the impact of the COVID-19 pandemic on women’s education, occupations, and health in Bangladesh. The study focused on five SDGs which directly affect women’s livelihood and well-being, namely, no poverty, good health, quality education, gender equality, and decent work and economic growth. The authors emphasized objectivity and utilized clear and concise language to convey their findings accurately. Technical abbreviations were explained upon first use, and the paper adhered to commonly accepted style and formatting guidelines. Five Sustainable Development Goals (SDGs) having a direct impact on women’s livelihoods and well-being were chosen and analyzed. These goals are: poverty eradication, promoting good health and well-being, providing quality education, ensuring gender equality, and fostering decent work and economic growth. The study indicates that the current neoliberal market economy has not succeeded in protecting the world from a pandemic. Therefore, to establish a harmonious society centered on caring relationships between nature, humans, and society, it is essential to dismantle the present economic system.

In summary, the DPSIR model plays a key role in conducting environmental and economic sustainability assessments and studies in various fields. The model assists in addressing natural resource and environmental concerns, including groundwater resource management, and provides critical reference material for decision makers. It underscores the significance of China’s leaf rock gas sector’s potential for sustainable development and the Yellow River Basin’s rural areas’ efficient sustainable development, as well as the relevance of related factors. In the field of socio-economic development, the DPSIR model offers comprehensive analysis and response tactics regarding topics like managing urban land, reducing urban SO_2_ emissions, implementing global fire governance, and coping with global development and challenges during the COVID-19 pandemic. This underscores the broad applicability of the DPSIR model, which this study adopts for constructing our model.

### The sustainability of the sports sector

Most of the evaluations, programs and studies related to sustainability programs in the sports sector and industry are based on resource allocation and sustainable development of the industry. For example, Zou et al. [20] adopted the Structural Equation Modeling (SEM) to investigate the factors that influence audience loyalty on Chinese live sports platforms. The study examined these factors from the three levels: government, industry, and platforms. The findings revealed that the perceived value of the audience to the live broadcast platform directly affects their loyalty. Additionally, the functionality of the live broadcast platform functionality, the easiness of use and the quality of service also have a direct impact on the audience’s loyalty. The study’s findings provided a valuable insight into the sales and marketing plans of the sports industry, which in turn offered insights into the sustainability of the industry. Qin and Liu [21] analyzed the issue of allocating sports resources to colleges and universities in Shaanxi Province, China, through an empirical investigation. They also proposed countermeasures and methods for sharing sports resources in colleges and universities, such as Xi’an Jiaotong University and Xi’an University of Foreign Studies, which provided valuable insights into the sustainability of sports resources in higher education. Kadagi et al. [22] concluded that the sustainability development plan of the marine sports industry, which may cause obstacles to the local socio-cultural and economic environment, and the development of regional fisheries, and therefore it is important to conduct a comprehensive assessment of these obstacles in order to reduce the impact caused by the development of the marine sports industry through a survey study. Zhao et al. [23] constructed an environmental science model for sports tourism based on socio-economic benefits and ecological environmental protection, which solved the drawbacks of the traditional sports tourism development model that only focuses on the perspective of operation, development, and management while ignoring the benefits to the economic and social interests and ecological environmental protection, and put forward a proposal for the sustainable development of the resource integration and functional integration of sports tourism. Josa et al. [24] proposed a new method for the rigid structure of stadium rooftop buildings, which integrates value modeling and multi-criteria decision-making to construct an evaluation index system that provided an important reference for the sustainability and durability of stadiums in the use of the building. Bellver et al. [25] adopted the empirical analysis method to conduct a questionnaire survey on 374 college students majoring in Physical Activity and Sports Sciences (PASS). The study indicated that the conditions of effective creation and management of sustainable businesses, along with a high level of social and civic values, and the support of the surrounding environment, are significant factors in achieving sustainable entrepreneurial intentions among students of physical education.

With the above in mind, studies on sustainability involving the sports sector and industry have mostly focused on the sustainable management of the industry, resource development, resource allocation, and environmental construction. In short, they are each focused on their own areas and an integrated evaluation model is lacking. Accordingly, this study constructed an evaluation model for the sustainability of competitive sports using the DPSIR model as the theoretical basis. The aim was to address the following problems in competitive sports: (1) the imbalance of development among different regions, (2) the low level of market participation, (3) the slow progress of professionalization reform, and (4) the low level of scientific and technological empowerment. This model aims to provide a set of systematic and scientific decision-making reference standards for government departments, managers, and decision-makers in related industries to support them in establishing sustainable development plans for competitive sports.

## Research methods

**Fig 1:**
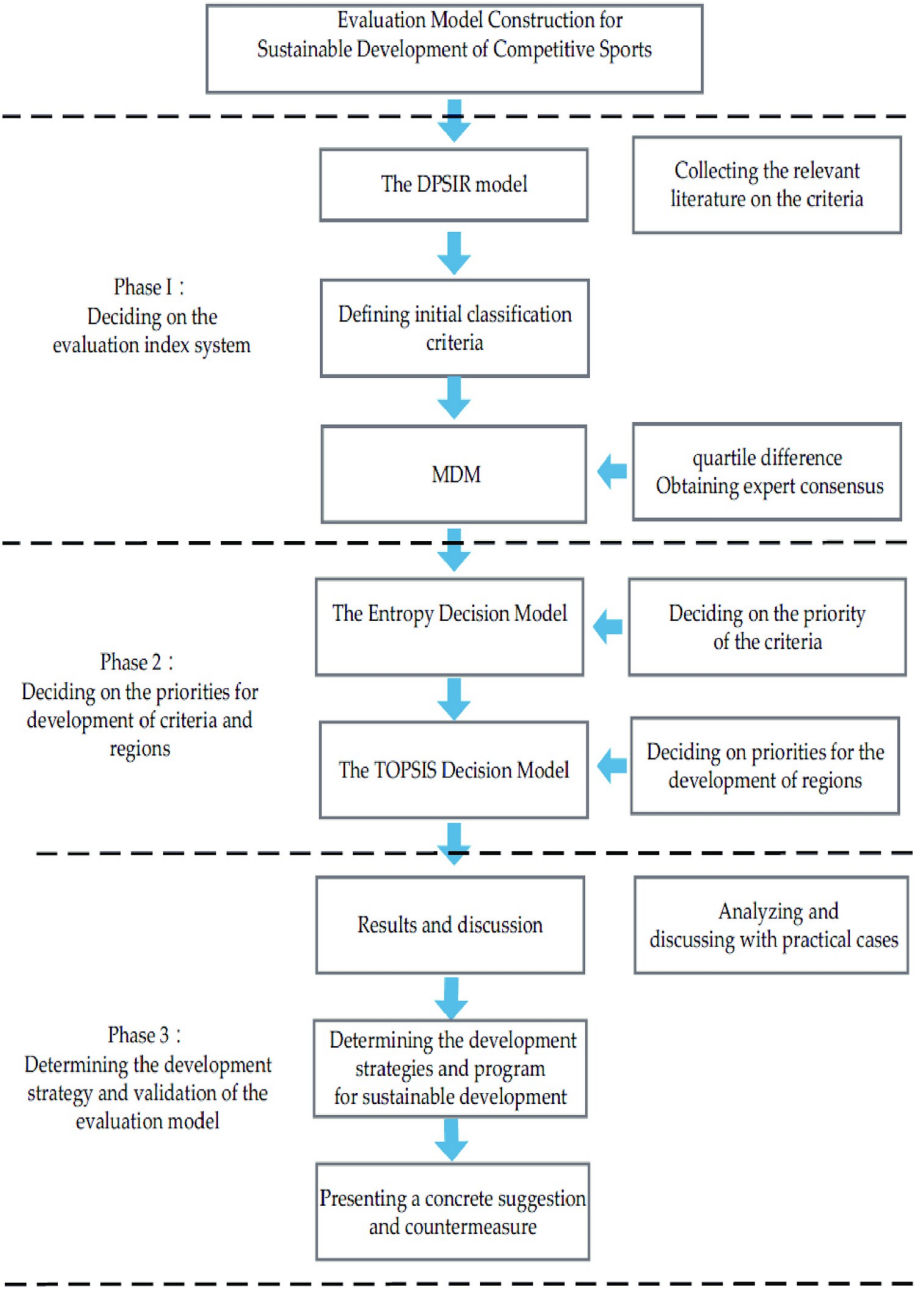
Research framework diagram.

This study constructed an evaluation index system for the sustainable development of competitive sports based on the DPSIR model. The construction of the evaluation model will be carried out in three stages. First, the evaluation index system is divided into five criteria based on the theoretical basis of the DPSIR model, after which the affiliation of each index at each level is determined by the modified Delphi method. The second stage involves the use of entropy to determine the weights of each criterion in the index system. The third stage used TOPSIS (Technique for Order Preference by Similarity to an Ideal Solution) to approximate the ideal point method in ranking the alternatives to verify the suitability of the evaluation index system. Finally, in response to the analysis results, specific measures for the development of competitive sports sustainability in the province were proposed. The framework of this paper is shown in Fig 1. The methodology of this study is described in the following sections.

### Constructing an evaluation index system based on the Delphi method and the modified Delphi method

The Delphi Method was pioneered by Helm and Dahlke developed the concept in the 1940s, which was later expanded upon by Gordon and the RAND Corporation [26]. The method has been applied to the military, technology, medical care, and market demand to make qualitative predictions, to avoid blindly following orders or submitting to authority without question. As a result, it has gained widespread acceptance. Cho and Lin [27]. pointed out that the implementation steps of the Delphi method can be organized into the following six steps: (1) drawing up the survey outline, (2) selecting experts, (3) conducting the first questionnaire survey, (4) conducting the second questionnaire survey, (5) conducting the third questionnaire survey, and (6) synthesizing opinions to form a consensus. The process of implementing the Delphi Method is detailed below. (1) First, a detailed outline of the questions to be answered by the expert is drawn up, and the expert is provided with relevant background material. (2) Anonymous, representative, and relevant experts in the field of competitive sports were selected to form a panel of experts and were provided with a prepared outline of the survey in accordance with the purpose of the survey. (3) The first questionnaire is an open-ended or semi-open-ended questionnaire that collects and summarizes the experts’ initial judgment and removes indexes on which there is no agreement. (4) To conduct a second questionnaire, which is structured similarly to the first questionnaire design, and send it to the experts again. This will allow the experts to compare their opinions with others and refine their judgments. (5) A third questionnaire will be conducted, identical to the second round. (6) A consensus among the experts is reached, and if there is no unanimous agreement, steps 4 and 5 are repeated until the experts’ opinions converge.

The traditional Delphi method has the following advantages. First, the opinions are anonymous, so experts are more likely to express their true opinions. Second, the opinions have equal weight, eliminating the influence of authority. Third, the experts do not have to be gathered in one place, but only communicate by correspondence, which makes implementation easier. Fourth, the final opinion of the expert group is more broadly representative and accurate. However, the traditional Delphi method has a major drawback, which is that it uses an unstructured questionnaire to extract the priorities and opinions of the experts, and it is time-consuming and complicated. Therefore, the scholars have proposed the modified Delphi method, which utilizes a structured (open-ended) questionnaire to amend the shortcomings of the traditional Delphi method. The Modified Delphi Method (MDM) is an expert survey method that applies a structured questionnaire instead of an open-ended questionnaire, thus enhancing the efficiency of the traditional Delphi method. Listone and Turoff [28] argued that the traditional Delphi method has the following limitations. (1) The results of the Delphi method are susceptible to interference by subjective expert judgment. (2) The process of implementing the Delphi method is likely to be influenced by the questionnaire’s distributor. (3) The Delphi method is time-consuming and labor-intensive, and it is not easy to monitor the progress. In this regard, this study applied the modified Delphi method to determine the comprehensive evaluation index system for the sustainability development of competitive sports based on the preliminary evaluation index system decided in the previous section.

### The prioritization of guidelines and regional development based on Entropy and TOPSIS

The Multiple Criteria Decision Making (MCDM) refers to the process of selecting from a finite or infinite set of alternatives that are mutually conflicting and incommensurable. Since the 1960s, Multi-Criteria Decision Making (MCDM) has been introduced as a normative method in the field of decision-making. It has gained widespread usage in various domains, including investment decision-making, management decision-making, and resource allocation. Many researchers and policy-makers have used MCDM to address complex social, economic, and scientific management problems that involve a multitude of factors. For example, Aksel et al. [29] employed the MCDM model to select suppliers for the aerospace and defense industry. Ofori et al. [30] applied the MCDM model to optimize decisions regarding renewable energy generation resources in Ghana. Kuttusi et al. [31] assessed the potential for glacial geoheritage development in the Yalnizam Mountains of northeastern Turkey through the use of MCMD. Dayarnab et al. [32] used the MCDM model for the selection of natural fibers as substrates for flexible sensors. Hamidah et al. [33] applied MCDM to establish a protocol for developing standardized weights for important plant areas in Malaysia. Shabani et al. [34] applied the MCDM technique to measure customer satisfaction in Tehran’s public transportation during the COVID-19 pandemic Mosetlhe et al. [35] presented a framework for microgrid configuration selection for water pumping applications in rural areas using the MCDM approach. The MCDM method includes Entropy, TOPSIS, ANP, AHP, GRA, ELECTRE, COPRAS, WASPAS, SECA, CODAS, SWARA II, MEREC and EDAS etc., and each of these methods has its own advantages and disadvantages in different application scenarios. And this research is to use entropy to combine TOPSIS on the grounds that entropy is an objective weighting method, which is based on the principle of using the concept of entropy value to determine the relative weights among the indicators. This method first evaluated the weight of each indicator and indicated the degree of influence of each indicator in the overall decision process by calculating its entropy values. The individual entropy values were then compared to determine the relative weight relationship between them. Therefore, when the entropy value was used to calculate the weights of the indicators, if the entropy value of an indicator was higher, then the weight of that indicator was greater, so that the different importance between different indicators could be distinguished. The entropy method calculates its entropy weights from the given raw data, without the interference of individual subjective factors. This makes the index weights more objective and ensures the scientific nature of the evaluation results [36]. Furthermore, TOPSIS’ computational process is relatively simple, computationally efficient, easily and quickly implemented in applications, and broadly adaptable to different data types and decision environments, including comprehensive evaluation of qualitative and quantitative data. TOPSIS is a widely used method for multi-criteria decision analysis. Finally, TOPSIS provides a clear and consistent framework for evaluating and comparing different options, thus increasing the transparency of the decision-making process. Overall, TOPSIS has been widely used in the field of multi-criteria decision analysis due to its many advantages such as comprehensiveness, intuitiveness, efficiency, practicality and adaptability [37, 38]. Given this, the purpose of combining Entropy and TOPSIS in this study was to use Entropy to determine the weights of the indicators to be evaluated and TOPSIS to rank the objects to be evaluated in the final order. The combination of these two methods increased the transparency and rationality of the decision-making process, improved the accuracy of the decision, balanced objectivity and subjectivity, reduced the impact of data uncertainty and bias, and had a wider scope of application [39].

According to the previous studies, Entropy and OPSIS methods have been proved to be relatively effective and fast problem solving, important in solving management development strategy problems, and investment project evaluation problems Cho and Lin. [27] The construction of evaluation models with Entropy and TOPSIS methods has been widely adopted in several fields such as, Zhang et al. [40] used Entropy and TOPSIS methods to evaluate the intelligence level of 15 new first-tier cities in China. Li et al. [41] evaluated the suitability level for regional shallow geothermal energy implementation using Entropy and TOPSIS methods. Wu et al. [42] assessed the operational safety of urban railway stations using Entropy and TOPSIS methods. Xu et al. [43] assessed the level of sustainable development of urban agglomerations in the Yangtze River Delta region using Entropy and TOPSIS methods Li, et al. [44] valued the risk management priorities of the historic and cultural reserves in 31 provinces of China from the use of Entropy and TOPSIS methods. These studies suggested that the combination of the Entropy and TOPSIS methods has a wide range of promising applications in multi-criteria decision-making. It is therefore scientifically reasonable to adopt this model to evaluate the sustainable development level of competitive sports in Chinese provinces. This study constructed the decision-making model of Entropy and TOPSIS on the basis of previous research, aiming to construct the evaluation index system of sustainable development level of provincial competitive sports in a scientific and reasonable way. The following section provides a detailed explanation of the construction process for the Entropy and TOPSIS models.

#### Entropy

The entropy is one of the highly used evaluation models in the field of MCDM (Multiple Criteria Decision Making). The entropy is an objective measure used to estimate the amount of information contained in each segment of data and calculated to determine the index weighting. The concept of entropy was originally developed as a metric to quantify the “messiness” of a system. The lower the level of molecular disorder in a given state of a system, the more aligned the molecules are. The more aligned the molecules are and the closer they are to perfect crystals, the greater the weights are. The more irregular the molecular arrangement, the higher the degree of molecular disorder. Entropy references weights to determine the relative weights between attributes. First, the weights of each attribute were calculated for each alternative to determine the extent to which the information possessed by the attribute could influence the overall decision outcome. Then the weights of each criterion were compared and the weights between them were calculated.

The process of the Entropy calculation is as follows Cho and Lin [27]:

Step 1: Establishing a decision matrix *D*
  The *D* decision matrix is as follows:
  $$\begin{array}{l} \;\;\;\;\;\;\;\;\;\;\;\;\;\;\;\;\;\;\;\;\;\;\;\;\;\;C_{1}\;\;\;\;\;\;\;C_{2}\;\;\begin{array}{c} \;\;\;\;\;\;\;\cdots \end{array}\;\;\begin{array}{c} \;\;\;\;\;\cdots \;\;\;\;C_{j}\;\; \end{array}\;\;\;\;\;C_{n}\\ D=\begin{array}{cc} \begin{array}{c} A_{1}\\ A_{2}\\ \vdots \\ A_{i}\\ \vdots \\ A_{m} \end{array} & \left[\begin{array}{cccccc} C_{11} & C_{12} & \cdots & \cdots & C_{1j} & C_{1n}\\ C_{21} & C_{22} & \cdots & \cdots & C_{2j} & C_{2n}\\ \vdots & \vdots & \cdots & \vdots & \vdots & \vdots \\ C_{i1} & C_{i2} & \vdots & \vdots & C_{ij} & C_{in}\\ \vdots & \vdots & \cdots & \vdots & \vdots & \vdots \\ C_{m1} & C_{m2} & \cdots & \cdots & C_{mj} & C_{mn} \end{array}\right] \end{array} \end{array}$$ (1)
  In the decision matrix *D*, *A*_1_, *A*_2_, *A*_3_, …, *A*_*m*_ denotes the number of experts; *C*_1_, *C*_2_, *C*_3_, …, *C*_*n*_ denotes the decision criteria.
  *t* and 10 being very important.
Step 2: Determining the results of the target attribute *P*_*ij*_
  The equation for the target attribute *P*_*ij*_ can be expressed as:
  $$\begin{array}{c} P_{ij}=\frac{C_{ij}}{\sqrt{\sum_{j=1}^{n}C_{ij}^{2}}} \end{array}$$ (2)
  and, *i* = 1, …, *m*; *j* = 1, …, *n*.
Step 3: Identifying *E*_*j*_
  Setting the weights of the target attributes to measure the results, and the range of weights should be between 0 ≤ *E*_*j*_ ≤ 1, as *k* value is a normal number.

$k=\frac{1}{lnm}$ , (*m* is the number of experts involved in the decision).
  $$\begin{array}{c} E_{j}=-K\sum_{i=1}^{m}P_{ij}\text{ln}P_{ij},\forall j \end{array}$$ (3)
Step 4: Defining *d*_*j*_
  The resultant rank of the target attribute is decentralised.
  $$\begin{array}{c} d_{j}=1-E_{j},\forall j \end{array}$$ (4)
Step 5: Confirming *W*_*j*_
  The expected comparative equalisation weights are as follows:
  $$\begin{array}{c} W_{j}=\frac{d_{j}}{\sum_{j=1}^{n}d_{j}},\forall j \end{array}$$ (5)

#### TOPSIS

The MCDM consists of a number of methods such as Entropy, TOPSIS, AHP, ANP, ELECTRE, GRA ……, etc., each of which has its own advantages and disadvantages in terms of use. From the past researches, it has been pointed out that the use of Entropy and TOPSIS is an important method to solve the problem efficiently and quickly when facing the evaluation of investment projects and management development strategies [31]. The Technique for Order Preference by Similarity to Ideal Solution (TOPSIS) method was first proposed by Hwang and Yoon in 1981 [45]. This method is used by the decision-maker to evaluate the relative advantages and disadvantages by specifying the positive and negative ideal solutions, and then comparing and measuring them on the basis of the positive ideal points to calculate the distance between the evaluation solution and the positive ideal solution. The main strength of the TOPSIS method is that the relative distance from the ideal solution is used to rank the advantages and disadvantages of the solutions, which avoids the difficulty of evaluating the solutions that are the closest to the positive ideal solution or the farthest from the negative ideal solution, and therefore not comparable.

The steps for the solution are as follows [8, 27, 46].

Step 1: Establishing a formal assessment matrix.
  A standardized assessment matrix was created with the following formula:
  $$\begin{array}{c} r_{ij}=\frac{C_{ij}}{\sqrt{\sum_{i=1}^{m}X_{ij}^{2}}} \end{array}$$ (6)
  where *i* is the alternative in the regularisation matrix and *j* is the evaluation criterion, *X*_*ij*_ is the evaluation value of alternative *i* under criterion *j*.
  Step 2: Building a regularised weight matrix
he regularised evaluation matrix, namely:
  $$\begin{array}{c} V=[\begin{array}{cccc} V_{11} & V_{12} & \cdots & V_{1n}\\ V_{21} & V_{22} & \cdots & V_{2n}\\ \vdots & \vdots & \ddots & \vdots \\ V_{m1} & V_{m2} & \cdots & V_{mn} \end{array}]=[\begin{array}{cccc} w_{1}r_{11} & w_{2}r_{12} & \cdots & w_{n}r_{1n}\\ w_{1}r_{21} & w_{2}r_{22} & \cdots & w_{n}r_{2n}\\ \vdots & \vdots & \ddots & \vdots \\ w_{1}r_{m1} & w_{2}r_{m2} & \cdots & w_{n}r_{mn} \end{array}] \end{array}$$ (7)
Step 3: Determining the positive and negative ideal solutions
  The positive and negative ideal solutions are deduced from the normalised weighting matrices, which are calculated by means of Eqs (8) and (9):
  $$\begin{array}{c} A^{+}=\{\left(\text{max}V_{ij}|j\in J\right),\left(\text{min}V_{ij}|j\in J^{\prime }\right),i=1,2,\cdots ,m\} \end{array}$$ (8)
  $$\begin{array}{c} A^{-}=\{(\text{min}V_{ij}|j\in J),(\text{max}V_{ij}\left|j\in J^{\prime }\right),i=1,2,\cdots ,m\} \end{array}$$ (9)
  where *J* denotes the criterion related to the profit or benefit and *J*′ denotes the criterion related to the cost or loss.
Step 4: Defining the gap between the ideal and the negative ideal solution for each scenario
  The Separation Measure is used to calculate the gap between the positive and negative ideal solutions respectively.
  The distance $S_{i}^{+}$ of the positive ideal solution is given by the following calculation:
  $$\begin{array}{c} S_{i}^{+}=\sqrt{\sum_{j=1}^{n}{\left(V_{ij}-V_{j}^{*}\right)}^{2},}\;i=1,2,\cdots ,m \end{array}$$ (10)
  The distance to the negative ideal solution A is calculated as follows:
  $$\begin{array}{c} S_{i}^{-}=\sqrt{\sum_{j=1}^{n}{\left(V_{ij}-V_{j}^{-}\right)}^{2},}\;i=1,2,\cdots ,m \end{array}$$ (11)
Step 5: Determining the relative closeness of the ideal solution $C_{i}^{*}$
  The relative proximity of the solution options to the ideal solution is calculated, and if the value is closer to 1, it means that the evaluated solution is closer to the ideal solution.
  $$\begin{array}{c} C_{i}^{*}=\frac{S_{i}^{-}}{S_{i}^{+}+S_{i}^{-}} \end{array}$$ (12)
Step 6: Sorting the calculated values in order of magnitude and select the best option.

## The construction of the evaluation model

This study aimed to construct an evaluation index system for the sustainability development of competitive sports relying on the DPSIR model. The construction of the evaluation model will be carried out in three stages, the main elements of which are detailed below.

### Phase 1: The evaluation index system based on DPSIR and the Delphi decisions

Step 1: Collecting the relevant literature and making preliminary organizations
  Firstly, the five guidelines based on the DPSIR theory are identified in this study, and a total of 150 related literature are collected based on these guidelines. Subsequently, after collating and summarizing the literature, the duplicated indexes were deleted and merged, and 30 sub-criteria were finally extracted. The data collection period spanned from October 2022 to December 2022.
Step 2: Defining a preliminary system of evaluation indexes and amending the Delphi questionnaire
  Three experts were invited to test the content validity of the scale in this study by using the five aforementioned criteria and 30 sub-criteria to design the Modified Delphi Questionnaire elicitation scale. Three issues that were not representative were deleted in accordance with the recommendations of the experts. Eventually, 24 sub-norms were identified, and a preliminary system of evaluation indicators and a questionnaire were developed. The implementation date is from December 2022 to January 2023, a total of one month.
Step 3: Developing a survey outline to determine a preliminary system of the evaluation indexes
  Based on the preliminary evaluation indicator system decided in step 2 above, the evaluation index system was divided into five evaluation criteria of DPSIR and 24 evaluation sub-criteria. Initially, there are 7 sub-criteria for Drive, 3 sub-criteria for Pressure, 4 sub-criteria for State, 4 sub-criteria for Impact, and 6 sub-criteria for Response. This study utilized the Likert Scale to assess the significance of each index, with a rating of 7 indicating high importance and a rating of 1 indicating low importance. A structured questionnaire was developed based on these rules, and after the Delphi questionnaire was revised by the experts, the definitions and categorization of the indicators were gradually revised according to the experts’ opinions until a consensus was reached.
Step 4: Selecting the expert group and implementing the questionnaire
  Murry and Hammons [47] considered that the Delphi method requires a group of experts consisting of more than ten people. A total of 20 experts were invited to form the expert panel for this study, taking into account both feasibility and workload. The selection of the expert panel mainly includes the following types: firstly, experts and scholars engaged in the research of competitive sports and elite sports both domestically and internationally; secondly, departmental leaders with extensive experience in the administrative management of competitive sports; and thirdly, coaches and athletes actively involved in the practice of competitive sports. Under these three aspects, 40% of the experts were from academia, 30% from the industry, and 30% from leaders of sports administrative bodies. Their research areas encompass a wide range of competitive sports, sports management, social sports, mass sports, and management science. Among them, there are 8 teachers who are mainly engaged in the research of competitive sports, and all of them have the title of associate professor or above; there are 6 experts from the sports authorities, who are mainly from sports administrative departments, and their main functional business is the management of competitive sports; 6 representatives are from the leading edge of competitive sports practice, who are primarily involved in coaching and refereeing in competitive sports.
Step 5: Soliciting questionnaires and determining the consistency of the experts’ opinion.
  This study conducted three rounds of the Modified Delphi questionnaire, which were delivered in person, by email, and by WeChat. Expert questionnaires are collated and analyzed before proceeding to the next round. The questionnaire survey was performed in March-April 2023, with three rounds of the Modified Delphi questionnaire.
Step 6: Deciding on a system of evaluation indexes
  The study employed the interquartile deviation as a method for examining the distribution of expert opinions, which is the difference between the upper quartile and the lower quartile of the distribution of expert opinions. The smaller the interquartile range, the more centered the views of the expert group are; the larger the interquartile range, the more divergent the views of the expert group are. If the interquartile variance is less than 0.6, then the index has a high degree of agreement among the expert group, and the index is retained. If the value is between 0.6 and 1.0, then there is a medium level of agreement and the index will be retained; if the index is greater than 1.0, then there is no agreement in the expert group on the index and it will be deleted. Finally, after two rounds of revising the Modified Delphi questionnaire, “China’s Competitive Sports Sustainable Development Evaluation Index System” is classified into five dimensions (criteria) and 24 sub-criteria, as shown in Table 1.

**Table 1:** The evaluation index system for the sustainable development of competitive sports in China.

Criteria	Sub-criteria	Unit	Attributes
*C*_1_: Drive (D)	*SC*_1_: Gross domestic product (GDP)	Billions of yuan	positive
	*SC*_2_: Total population at year-end	10,000 people	positive
	*SC*_3_: Per capita disposable income of the whole population	yuan	positive
	*SC*_4_: Research and development full-time equivalent staff	man-year	positive
	*SC*_5_: The investment in fixed assets in culture, sports and recreation	Billions of yuan	positive
	$SC_{6}^{*}$ : The number of employees in the sports system	people	positive
	$SC_{7}^{*}$ : The number of fitness activity centers	number	positive
*C*_2_: Pressure (P)	*SC*_8_: Crude birth rate	%	positive
	*SC*_9_: Unemployment rate of the urban population	%	negative
	*SC*_10_: The Green Coverage Rate in Developed Areas	%	positive
*C*_3_: State (S)	$SC_{11}^{*}$ : The number of the outstanding athletes of the sports teams	people	positive
	$SC_{12}^{*}$ : The national physique qualified rate	%	positive
	$SC_{13}^{*}$ : The number of the sports social organization	number	positive
	$SC_{14}^{*}$ : sports lottery sales	10,000 yuan	positive
*C*_4_: Impact (I)	*SC*_15_: The growth value of the tertiary industry	Billions of yuan	positive
	*SC*_16_: The Employment in culture, sports and recreation	10,000 people	positive
	*SC*_17_: Mortality rate	%	negative
	*SC*_18_: forest coverage rate	%	positive
*C*_5_: Response (R)	$SC_{19}^{*}$ : The number of the level athletes developed	people	positive
	$SC_{20}^{*}$ : The number of the athletic reserves	people	positive
	$SC_{21}^{*}$ : The number of the youth sports clubs	number	positive
	$SC_{22}^{*}$ : The number of National Physical Fitness Monitoring Stations	number	positive
	$SC_{23}^{*}$ : The number of National Fitness Trail Projects	number	positive
	$SC_{24}^{*}$ : The number of sports research projects	number	positive

Note:

* Indicators are from the China Sports Statistics Yearbook [013-2020] [48], other indicators are from the China Statistics Yearbook [013-2020] [49]

### Phase 2: Determining evaluation index weights based on Entropy

According to the evaluation index system constructed in Table 1, this study designed the questionnaire solicitation form to determine the weights of individual sub-criteria by the Entropy. The expert group invited was the aforementioned 20 experts in the revision of the Modified Delphi to be solicited, with each expert being consulted on a ten-scaled scale, and the measurement period was from 16th to 29th, April, 2023. After the scale was recovered, the Entropy model was used to determine the weights of each criterion and the analysis process was described below.

Step 1: Constructing the decision matrix *D* (Eq 1) and obtaining the target attribute *P*_*ij*_.
  This paper constructed a D-matrix based on the importance ratings of 20 experts for each metric, using a 10-point scale. A score of 1 indicates very unimportant and a score of 10 indicates very important. As shown in Table 2, C11 is equal to 8, which means that Expert 1 rated the importance of “SC1: Gross Domestic Product” with a score of 8. C21 equals 7, indicating that the second expert rated the importance of “SC1” as a score of 7. The rating of “SC1” by Expert 3 is 7 in C31 and 8 in C41 by Expert 4. Similarly, 20 experts evaluated each of the 24 indicators to construct the decision matrix *D*, and then the results of the target attributes were inputted into Eq (2) to obtain *P*_*ij*_, as shown in Table 3.
Step 2: Determining the weight of each criterion.
  Then the results based on Table 3 are substituted into Eqs (7), (8) and (9) to obtain the values of entropy measure *E*_*j*_, rank *d*_*j*_, and weight *W*_*j*_ respectively, as shown in Table 4.

**Table 2:** Decision matrix from the experts’ judgement.

	*C*_1_: Drive (D)							*C*_2_: Pressure (P)			*C*_3_: State (S)				*C*_4_: Impact (I)				*C*_5_: Response (R)					
	*SC*_1_	*SC*_2_	*SC*_3_	*SC*_4_	*SC*_5_	*SC*_6_	*SC*_7_	*SC*_8_	*SC*_9_	*SC*_10_	*SC*_11_	*SC*_12_	*SC*_13_	*SC*_14_	*SC*_15_	*SC*_16_	*SC*_17_	*SC*_18_	*SC*_19_	*SC*_20_	*SC*_21_	*SC*_22_	*SC*_23_	*SC*_24_
*EV*_1_	8	5	8	9	8	9	8	6	6	6	9	7	8	9	8	8	5	5	8	8	8	7	9	8
*EV*_2_	7	7	7	8	5	8	7	6	6	7	7	6	5	8	7	8	6	6	7	7	6	5	8	7
*EV*_3_	7	6	8	6	7	8	9	7	7	6	5	6	7	8	5	6	7	7	6	9	6	7	8	9
*EV*_4_	8	8	6	7	9	9	6	5	8	8	9	7	5	9	6	6	5	8	9	6	7	5	8	9
*EV*_5_	8	6	9	9	6	5	7	6	7	6	9	7	6	5	7	9	6	7	9	9	7	6	5	7
*EV*_6_	7	6	8	9	9	8	8	6	6	6	7	7	9	8	8	7	6	6	6	9	7	9	8	8
*EV*_7_	9	7	8	8	8	8	9	8	6	7	6	6	8	8	6	8	8	6	9	6	9	8	8	9
*EV*_8_	9	5	9	8	9	7	6	9	8	5	9	6	6	7	5	6	9	6	9	9	8	6	7	6
*EV*_9_	9	6	8	8	9	9	8	5	9	6	7	5	8	9	5	7	5	9	6	9	9	8	9	9
*EV*_10_	7	7	8	9	6	9	7	7	6	5	8	7	8	9	7	5	5	6	9	8	9	6	8	9
*EV*_11_	9	8	8	7	7	9	6	5	8	8	9	6	9	9	6	6	5	6	8	9	9	5	9	6
*EV*_12_	7	6	9	9	6	5	7	6	7	6	9	6	6	5	7	8	5	7	9	9	9	8	5	7
*EV*_13_	7	6	8	7	7	8	8	6	6	6	8	7	9	8	8	7	5	6	9	9	9	9	8	8
*EV*_14_	8	7	6	9	8	8	9	8	6	7	6	6	8	8	5	8	5	6	7	6	6	9	8	8
*EV*_15_	9	7	9	9	6	7	6	9	8	5	9	8	9	7	5	9	5	6	9	9	9	6	7	9
*EV*_16_	9	6	7	7	6	9	8	5	9	6	9	5	6	9	6	7	5	9	9	9	9	9	9	8
*EV*_17_	7	7	9	9	7	9	7	7	6	5	9	7	9	9	7	9	7	6	9	9	7	9	9	7
*EV*_18_	9	7	9	9	9	7	6	9	8	5	9	8	6	9	6	6	9	8	9	9	9	6	7	9
*EV*_19_	9	6	9	5	9	9	8	5	9	6	9	5	6	9	8	7	5	9	9	9	9	6	9	8
*EV*_20_	8	5	8	7	6	9	7	7	6	5	8	7	6	9	7	5	7	6	9	8	7	6	9	9

**Table 3:** The results of the target attribute *P*_*ij*_ obtained by Entropy.

	*C*_1_: Drive (D)							*C*_2_: Pressure (P)			*C*_3_: State (S)				*C*_4_: Impact (I)				*C*_5_: Response (R)					
	*SC*_1_	*SC*_2_	*SC*_3_	*SC*_4_	*SC*_5_	*SC*_6_	*SC*_7_	*SC*_8_	*SC*_9_	*SC*_10_	*SC*_11_	*SC*_12_	*SC*_13_	*SC*_14_	*SC*_15_	*SC*_16_	*SC*_17_	*SC*_18_	*SC*_19_	*SC*_20_	*SC*_21_	*SC*_22_	*SC*_23_	*SC*_24_
*EV*_1_	0.050	0.039	0.050	0.057	0.054	0.056	0.054	0.045	0.042	0.050	0.056	0.054	0.056	0.056	0.062	0.056	0.042	0.037	0.048	0.048	0.050	0.050	0.057	0.050
*EV*_2_	0.043	0.055	0.043	0.050	0.034	0.050	0.048	0.045	0.042	0.058	0.043	0.047	0.035	0.049	0.054	0.056	0.050	0.044	0.042	0.042	0.038	0.036	0.051	0.044
*EV*_3_	0.043	0.047	0.050	0.038	0.048	0.050	0.061	0.053	0.049	0.050	0.031	0.047	0.049	0.049	0.039	0.042	0.058	0.052	0.036	0.054	0.038	0.050	0.051	0.056
*EV*_4_	0.050	0.063	0.037	0.044	0.061	0.056	0.041	0.038	0.056	0.066	0.056	0.054	0.035	0.056	0.047	0.042	0.042	0.059	0.055	0.036	0.044	0.036	0.051	0.056
*EV*_5_	0.050	0.047	0.056	0.057	0.041	0.031	0.048	0.045	0.049	0.050	0.056	0.054	0.042	0.031	0.054	0.063	0.050	0.052	0.055	0.054	0.044	0.043	0.032	0.044
*EV*_6_	0.043	0.047	0.050	0.057	0.061	0.050	0.054	0.045	0.042	0.050	0.043	0.054	0.063	0.049	0.062	0.049	0.050	0.044	0.036	0.054	0.044	0.064	0.051	0.05
*EV*_7_	0.056	0.055	0.050	0.050	0.054	0.050	0.061	0.061	0.042	0.058	0.037	0.047	0.056	0.049	0.047	0.056	0.067	0.044	0.055	0.036	0.057	0.057	0.051	0.056
*EV*_8_	0.056	0.039	0.056	0.050	0.061	0.044	0.041	0.068	0.056	0.041	0.056	0.047	0.042	0.043	0.039	0.042	0.075	0.044	0.055	0.054	0.050	0.043	0.044	0.038
*EV*_9_	0.056	0.047	0.050	0.050	0.061	0.056	0.054	0.038	0.063	0.050	0.043	0.039	0.056	0.056	0.039	0.049	0.042	0.067	0.036	0.054	0.057	0.057	0.057	0.056
*EV*_10_	0.043	0.055	0.050	0.057	0.041	0.056	0.048	0.053	0.042	0.041	0.050	0.054	0.056	0.056	0.054	0.035	0.042	0.044	0.055	0.048	0.057	0.043	0.051	0.056
*EV*_11_	0.056	0.063	0.050	0.044	0.048	0.056	0.041	0.038	0.056	0.066	0.056	0.047	0.063	0.056	0.047	0.042	0.042	0.044	0.048	0.054	0.057	0.036	0.057	0.038
*EV*_12_	0.043	0.047	0.056	0.057	0.041	0.031	0.048	0.045	0.049	0.050	0.056	0.047	0.042	0.031	0.054	0.056	0.042	0.052	0.055	0.054	0.057	0.057	0.032	0.044
*EV*_13_	0.043	0.047	0.050	0.044	0.048	0.050	0.054	0.045	0.042	0.050	0.050	0.054	0.063	0.049	0.062	0.049	0.042	0.044	0.055	0.054	0.057	0.064	0.051	0.050
*EV*_14_	0.050	0.055	0.037	0.057	0.054	0.050	0.061	0.061	0.042	0.058	0.037	0.047	0.056	0.049	0.039	0.056	0.042	0.044	0.042	0.036	0.038	0.064	0.051	0.050
*EV*_15_	0.056	0.055	0.056	0.057	0.041	0.044	0.041	0.068	0.056	0.041	0.056	0.062	0.063	0.043	0.039	0.063	0.042	0.044	0.055	0.054	0.057	0.043	0.044	0.056
*EV*_16_	0.056	0.047	0.043	0.044	0.041	0.056	0.054	0.038	0.063	0.050	0.056	0.039	0.042	0.056	0.047	0.049	0.042	0.067	0.055	0.054	0.057	0.064	0.057	0.050
*EV*_17_	0.043	0.055	0.056	0.057	0.048	0.056	0.048	0.053	0.042	0.041	0.056	0.054	0.063	0.056	0.054	0.063	0.058	0.044	0.055	0.054	0.044	0.064	0.057	0.044
*EV*_18_	0.056	0.055	0.056	0.057	0.061	0.044	0.041	0.068	0.056	0.041	0.056	0.062	0.042	0.056	0.047	0.042	0.075	0.059	0.055	0.054	0.057	0.043	0.044	0.056
*EV*_19_	0.056	0.047	0.056	0.031	0.061	0.056	0.054	0.038	0.063	0.050	0.056	0.039	0.042	0.056	0.062	0.049	0.042	0.067	0.055	0.054	0.057	0.043	0.057	0.050
*EV*_20_	0.050	0.039	0.050	0.044	0.041	0.056	0.048	0.053	0.042	0.041	0.050	0.054	0.042	0.056	0.054	0.035	0.058	0.044	0.055	0.048	0.044	0.043	0.057	0.056

**Table 4:** The results of entropy measure (*E*_*j*_), rank (*d*_*j*_) and weight (*W*_*j*_) obtained by Entropy.

	*C*_1_: Drive (D)							*C*_2_: Pressure (P)			*C*_3_: State (S)				*C*_4_: Impact (I)				*C*_5_: Response (R)					
	*SC*_1_	*SC*_2_	*SC*_3_	*SC*_4_	*SC*_5_	*SC*_6_	*SC*_7_	*SC*_8_	*SC*_9_	*SC*_10_	*SC*_11_	*SC*_12_	*SC*_13_	*SC*_14_	*SC*_15_	*SC*_16_	*SC*_17_	*SC*_18_	*SC*_19_	*SC*_20_	*SC*_21_	*SC*_22_	*SC*_23_	*SC*_24_
*E*_*jj*_	0.998	0.997	0.998	0.996	0.995	0.996	0.997	0.993	0.996	0.996	0.996	0.997	0.994	0.996	0.995	0.995	0.992	0.995	0.997	0.997	0.996	0.993	0.996	0.997
*d*_*j*_	0.002	0.003	0.002	0.004	0.005	0.004	0.003	0.007	0.004	0.004	0.004	0.003	0.006	0.004	0.005	0.005	0.008	0.005	0.003	0.003	0.004	0.007	0.004	0.003
*W*_*j*_	0.019	0.029	0.022	0.037	0.052	0.041	0.031	0.067	0.041	0.037	0.041	0.029	0.062	0.040	0.045	0.049	0.076	0.048	0.033	0.030	0.036	0.069	0.039	0.026

### Phase 3: Prioritization of districts based on TOPSIS decisions

Taking the 31 provinces, autonomous regions and municipalities directly under the central government as the object of study, this paper uses TOPSIS to prioritise the 31 provinces according to the “Evaluation Index System for Sustainable Development of Competitive Sports in China” constructed in this study. The analysis process is explained below.

Step 1: Establishing a decision matrix
  The data collection in this study was based on the 24 sub-criteria in Table 1, and the sources of data collection are the China Statistical Yearbook and Statistical Yearbook of sports undertakings. The example is depicted as a comprehensive evaluation result for the years 2013—2020, with the data for each region shown in Table 5. The respective evaluation results for each year will be presented in phase 3.
Step 2: Establishing the regularisation and weighting matrix
  Firstly, the matrix was normalised using Eq (6) based on the raw data (decision matrix) in Table 5. After that, the matrix is weighted with Eq (7). The weights of the indexes are determined by Entropy and the result obtained is the decision of the weighting matrix and the results are shown in Table 6.
Step 3: Deciding on a positive ideal solution versus a negative ideal solution
  The results obtained from the weighting matrix of Table 6 are applied to obtain the positive and negative ideal solutions according to Eqs (8) and (9).
Step 4: Identifying $S_{i}^{+}$, $S_{i}^{-}$ and $C_{i}^{*}$
  The adjusted values of the assessment matrix are then substituted into Eqs (7), (10) and (11) to obtain $S_{i}^{+}$ and $S_{i}^{-}$. The result of $C_{i}^{*}$ (the relative proximity) based on Eq (12) is the degree of the 31 regions’ strengths and weaknesses in the sustainability of competitive sports development from 2013 to 2020, as shown in Table 7.

**Table 5:** The statistical values of the indexes from 2013 to 2020 by regions in China.

Criteria Regions	*C*_1_: Drive (D)							*C*_2_: Pressure (P)			*C*_3_: State (S)				*C*_4_: Impact (I)				*C*_5_: Response (R)					
	*SC*_1_	*SC*_2_	*SC*_3_	*SC*_4_	*SC*_5_	*SC*_6_	*SC*_7_	*SC*_8_	*SC*_9_	*SC*_10_	*SC*_11_	*SC*_12_	*SC*_13_	*SC*_14_	*SC*_15_	*SC*_16_	*SC*_17_	*SC*_18_	*SC*_19_	*SC*_20_	*SC*_21_	*SC*_22_	*SC*_23_	*SC*_24_
Shanghai	29830.09	2442.25	57144.56	90661.25	88.64	4846.13	17.50	2.21	3.86	37.91	942.88	97.10	793.75	406101.67	20687.57	6.35	5.29	11.98	1819.13	10850.25	391.75	247.88	1008.88	91.00
Beijing	51011.81	2168.96	55385.90	50971.75	146.20	5426.88	88.13	3.30	1.50	48.42	1046.88	91.60	577.63	632370.46	22181.72	18.50	5.17	38.81	1764.88	23544.25	180.75	286.63	976.75	20.25
Tianjin	16263.66	1476.22	35417.96	63986.13	69.40	2794.25	15.25	1.35	3.53	36.66	812.25	93.00	349.50	333256.94	9148.90	2.23	5.65	10.70	1853.88	5528.50	66.63	62.13	897.00	6.63
Hebei	32830.29	7433.53	20926.30	76642.25	499.04	6049.88	22.38	5.06	3.52	41.71	999.38	84.10	1685.63	3455191.97	14329.73	5.28	6.49	25.25	2217.75	16116.25	265.75	186.38	3628.13	51.75
Shanxi	14690.83	3601.87	20001.61	30898.25	125.83	4472.88	9.25	4.04	3.28	41.10	563.5	93.00	1655.88	224958.88	7431.53	4.58	5.82	18.95	1582.00	8771.75	103.25	198.88	1529.38	27.50
Inner Mongolia	17378.85	2475.32	25291.11	23223.5	141.10	3560.50	46.75	2.51	3.67	39.61	734.88	85.90	1595.63	368166.34	7747.83	3.41	5.91	21.43	1455.00	12705.63	272.63	96.75	905.88	26.75
Liaoning	25737.74	4331.56	27043.53	54436.63	108.44	6909.75	45.13	-0.85	3.81	40.01	1820.00	89.70	1352.25	435777.84	12378.57	4.88	7.02	38.62	1843.63	15025.63	327.75	334.38	1285.25	4.75
Jilin	13718.35	2629.63	20831.23	18826.25	116.30	4355.13	10.75	-0.33	3.46	36.41	639.13	87.80	1092.63	319040.58	5612.81	3.54	6.27	40.68	950.38	9090.88	236.13	117.25	750.25	32.25
Heilongjiang	14970.6	3602.61	20603.28	25658.50	101.35	4966.63	17.13	-0.83	4.09	36.00	1340.13	87.80	1439.38	410652.33	7582.67	3.81	6.81	43.39	1016.63	9645.38	104.00	151.13	1794.38	14.50
Jiangsu	81412.43	8161.91	33933.43	458518.75	537.29	9149.25	72.63	1.94	3.03	42.96	1589.88	91.20	5576.25	1663645.99	40654.09	8.23	7.00	15.58	2475.13	20868.13	544.38	824.25	4707.63	146.25
Zhejiang	50255.34	5863.50	40835.13	356550.13	350.42	5481.38	26.50	4.76	2.80	41.00	1069.88	93.70	3285.38	1093488.09	26214.97	7.33	5.57	59.21	2613.00	21363.13	617.75	1645.88	3695.63	15.38
Anhui	27416.41	6122.48	21334.46	106543.75	230.33	4192.88	36.50	6.06	3.00	41.68	719.50	92.50	2142.50	495471.38	11940.27	3.44	6.22	27.95	1537.13	16249.13	489.88	108.13	2060.00	50.38
Fujian	31839.45	3950.75	29133.91	113214.63	465.23	4968.63	28.75	6.80	3.69	43.50	1116.00	88.30	2047.13	635633.31	13940.19	4.23	6.16	66.27	1435.13	23403.00	69.13	114.00	1277.88	21.75
Jiangxi	19798.45	4549.00	21346.34	52818.50	225.96	3621.50	32.50	6.43	3.26	45.05	570.25	91.60	1834.00	428744.07	8455.41	3.33	6.30	60.83	1293.25	15702.63	159.63	218.50	911.13	41.13
Shandong	67310.27	9958.76	25984.73	233816.25	808.50	10790.63	52.13	6.20	3.33	42.16	1332.75	91.50	3976.75	1412876.65	32264.08	7.08	7.05	16.93	4165.88	26317.88	171.00	412.63	4058.75	86.63
Henan	43320.72	9640.75	19539.15	132578.38	614.47	6499.13	29.63	4.85	3.03	39.40	552.50	91.50	2947.00	1044778.88	18570.25	7.51	6.99	22.49	2525.50	13847.88	386.13	392.63	2540.38	100.88
Hubei	34750.01	5855.31	22792.51	100065.75	678.93	6159.00	29.63	4.17	2.83	38.49	2315.75	94.20	1749.75	624152.22	16043.84	6.25	6.86	38.85	2272.00	18009.5	284.00	413.25	4108.00	126.00
Hunan	32944.93	6726.68	22432.75	93336.38	585.59	5519.75	19.38	4.96	3.70	40.20	671.13	84.30	1699.38	468553.05	13744.21	5.66	7.18	48.49	1665.13	14149.88	262.13	151.25	2967.63	16.00
Guangdong	86151.59	11480.75	32014.58	513473.75	307.9	10757.75	66.88	7.05	2.43	42.63	1700.00	95.90	2992.63	1488786.18	45553.34	11.96	4.56	52.11	3201.50	34925.00	567.88	432.38	2452.75	71.63
Guangxi	18421.10	4867.5	19262.23	19724.38	238.18	4168.75	24.13	7.34	2.78	39.16	881.63	86.90	1101.38	185390.06	8073.66	3.29	6.22	58.54	835.00	12679.13	189.25	252.75	1543.00	2.88
Hainan	4316.30	942.67	21819.61	2518.75	90.96	942.75	5.25	7.77	2.36	40.55	164.63	90.30	348.38	84686.08	2396.50	1.29	6.00	56.94	393.38	2634.13	35.88	60.38	436.38	2.00
Chongqing	18584.07	3082.69	23418.48	52945.25	152.35	1617.50	9.63	3.05	3.46	40.94	331.75	92.70	791.50	330022.22	9238.96	2.79	7.33	40.64	2133.38	15048.63	370.75	73.63	819.00	9.00
Sichuan	36300.50	8257.28	20034.35	69439.13	377.44	5711.38	10.88	2.88	3.88	39.91	1276.00	84.20	2439.88	472988.98	17278.72	5.93	7.11	36.27	2035.50	38052.00	304.38	115.63	2243.50	4.00
Guizhou	12806.67	3650.32	16199.87	18831.25	237.26	2346.75	7.38	6.23	3.30	38.41	223.38	81.50	1133.75	251731.71	6039.47	4.69	7.04	39.60	487.75	2152.13	38.75	42.00	559.63	5.63
Yunnan	16897.84	4731.59	17762.80	20264.13	247.47	4031.00	13.75	5.85	3.67	37.64	636.63	89.10	1598.38	630524.24	8072.51	3.65	6.65	51.91	827.75	10919.63	178.25	181.25	879.13	9.38
Xizang(Tibet)	1286.93	337.88	15044.91	180.50	30.57	793.25	16.50	10.35	2.68	36.70	183.00	76.40	27.25	77228.01	668.64	0.73	5.03	12.04	34.75	925.13	98.13	62.75	491.13	3.25
Shanxi	21183.60	3853.50	20066.56	45344.38	409.16	5734.25	39.75	3.76	3.33	40.03	843.50	87.20	1137.38	417599.41	8975.03	5.23	6.38	42.04	995.38	16707.63	87.13	185.13	424.75	150.63
Gansu	7567.06	2611.75	15531.19	10915.38	154.47	3433.63	31.25	5.01	2.58	32.96	684.50	90.60	881.13	254912.60	3869.76	2.65	6.59	11.30	751.25	7972.00	255.38	117.13	611.38	46.13
Qinghai	2606.98	588.75	18356.21	1754.25	66.62	1159.38	12.25	7.72	2.90	32.66	250.00	84.50	468.38	66464.67	1156.35	0.84	6.26	5.70	249.25	1285.25	141.13	63.00	349.00	3.38
Ningxia	3276.91	686.13	19967.86	6453.75	31.09	987.38	7.25	8.03	3.93	39.88	170.13	87.80	330.00	94064.68	1530.36	0.91	5.05	12.17	679.13	4796.38	192.25	50.88	359.50	1.63
Xinjiang	10885.49	2429.25	19050.48	6162.63	107.13	2691.88	13.38	8.38	2.69	38.70	591.88	82.50	502.13	223816.27	5289.28	2.86	4.72	4.47	792.63	12995.00	138.00	225.75	502.50	3.88

Note: For the names and units of each index SC1 to SC24, please refer to the definitions of the sub-criteria in Table 1.

**Table 6:** The results of the weighting matrix obtained from the TOPSIS.

Criteria Regions	*C*_1_: Drive (D)							*C*_2_: Pressure (P)			*C*_3_: State (S)				*C*_4_: Impact (I)				*C*_5_: Response (R)					
	*SC*_1_	*SC*_2_	*SC*_3_	*SC*_4_	*SC*_5_	*SC*_6_	*SC*_7_	*SC*_8_	*SC*_9_	*SC*_10_	*SC*_11_	*SC*_12_	*SC*_13_	*SC*_14_	*SC*_15_	*SC*_16_	*SC*_17_	*SC*_18_	*SC*_19_	*SC*_20_	*SC*_21_	*SC*_22_	*SC*_23_	*SC*_24_
Shanghai	0.0029	0.0024	0.0084	0.0038	0.0025	0.0068	0.0028	0.0049	0.0087	0.0063	0.0070	0.0058	0.0045	0.0033	0.0099	0.0092	0.0115	0.0027	0.0060	0.0035	0.0087	0.0079	0.0034	0.0074
Beijing	0.0050	0.0022	0.0081	0.0021	0.0041	0.0076	0.0143	0.0073	0.0034	0.0080	0.0077	0.0054	0.0032	0.0051	0.0106	0.0269	0.0113	0.0088	0.0058	0.0077	0.0040	0.0091	0.0033	0.0016
Tianjin	0.0016	0.0015	0.0052	0.0027	0.0019	0.0039	0.0025	0.0030	0.0080	0.0061	0.0060	0.0055	0.0020	0.0027	0.0044	0.0032	0.0123	0.0024	0.0061	0.0018	0.0015	0.0020	0.0030	0.0005
Hebei	0.0032	0.0074	0.0031	0.0032	0.0138	0.0085	0.0036	0.0112	0.0079	0.0069	0.0074	0.0050	0.0095	0.0279	0.0069	0.0077	0.0141	0.0057	0.0073	0.0053	0.0059	0.0059	0.0123	0.0042
Shanxi	0.0014	0.0036	0.0029	0.0013	0.0035	0.0063	0.0015	0.0090	0.0074	0.0068	0.0042	0.0055	0.0093	0.0018	0.0036	0.0066	0.0127	0.0043	0.0052	0.0029	0.0023	0.0063	0.0052	0.0022
Inner Mongolia	0.0017	0.0025	0.0037	0.0010	0.0039	0.0050	0.0076	0.0055	0.0083	0.0065	0.0054	0.0051	0.0090	0.0030	0.0037	0.0050	0.0129	0.0048	0.0048	0.0042	0.0061	0.0031	0.0031	0.0022
Liaoning	0.0025	0.0043	0.0040	0.0023	0.0030	0.0097	0.0073	0.0019	0.0086	0.0066	0.0135	0.0053	0.0076	0.0035	0.0059	0.0071	0.0153	0.0087	0.0060	0.0049	0.0073	0.0106	0.0044	0.0004
Jilin	0.0013	0.0026	0.0031	0.0008	0.0032	0.0061	0.0017	0.0007	0.0078	0.0060	0.0047	0.0052	0.0061	0.0026	0.0027	0.0051	0.0137	0.0092	0.0031	0.003	0.0052	0.0037	0.0026	0.0026
Heilongjiang	0.0015	0.0036	0.0030	0.0011	0.0028	0.0070	0.0028	0.0018	0.0092	0.0059	0.0099	0.0052	0.0081	0.0033	0.0036	0.0055	0.0148	0.0098	0.0033	0.0032	0.0023	0.0048	0.0061	0.0012
Jiangsu	0.0080	0.0081	0.0050	0.0192	0.0149	0.0129	0.0118	0.0043	0.0068	0.0071	0.0118	0.0054	0.0313	0.0134	0.0194	0.0119	0.0152	0.0035	0.0081	0.0068	0.0121	0.0262	0.0160	0.0118
Zhejiang	0.0049	0.0058	0.0060	0.0150	0.0097	0.0077	0.0043	0.0105	0.0063	0.0068	0.0079	0.0056	0.0185	0.0088	0.0125	0.0106	0.0121	0.0134	0.0086	0.0070	0.0137	0.0523	0.0126	0.0012
Anhui	0.0027	0.0061	0.0031	0.0045	0.0064	0.0059	0.0059	0.0134	0.0068	0.0069	0.0053	0.0055	0.0120	0.0040	0.0057	0.0050	0.0136	0.0063	0.0050	0.0053	0.0109	0.0034	0.0070	0.0041
Fujian	0.0031	0.0039	0.0043	0.0048	0.0129	0.0070	0.0047	0.0151	0.0083	0.0072	0.0083	0.0052	0.0115	0.0051	0.0067	0.0061	0.0134	0.0149	0.0047	0.0076	0.0015	0.0036	0.0043	0.0018
Jiangxi	0.0019	0.0045	0.0031	0.0022	0.0063	0.0051	0.0053	0.0142	0.0074	0.0074	0.0042	0.0054	0.0103	0.0035	0.0040	0.0048	0.0137	0.0137	0.0042	0.0051	0.0035	0.0069	0.0031	0.0033
Shandong	0.0066	0.0099	0.0038	0.0098	0.0224	0.0152	0.0084	0.0137	0.0075	0.0070	0.0099	0.0054	0.0223	0.0114	0.0154	0.0103	0.0154	0.0038	0.0136	0.0086	0.0038	0.0131	0.0138	0.0070
Henan	0.0042	0.0096	0.0029	0.0056	0.0170	0.0091	0.0048	0.0107	0.0068	0.0065	0.0041	0.0054	0.0166	0.0084	0.0089	0.0109	0.0152	0.0051	0.0083	0.0045	0.0086	0.0125	0.0086	0.0082
Hubei	0.0034	0.0058	0.0033	0.0042	0.0188	0.0087	0.0048	0.0092	0.0064	0.0064	0.0171	0.0056	0.0098	0.0050	0.0077	0.0091	0.0149	0.0088	0.0074	0.0059	0.0063	0.0131	0.014	0.0102
Hunan	0.0032	0.0067	0.0033	0.0039	0.0162	0.0078	0.0031	0.0110	0.0084	0.0066	0.0050	0.0050	0.0095	0.0038	0.0066	0.0082	0.0157	0.0109	0.0055	0.0046	0.0058	0.0048	0.0101	0.0013
Guangdong	0.0084	0.0115	0.0047	0.0216	0.0085	0.0151	0.0108	0.0156	0.0055	0.0070	0.0126	0.0057	0.0168	0.0120	0.0218	0.0174	0.0099	0.0118	0.0105	0.0114	0.0126	0.0137	0.0083	0.0058
Guangxi	0.0018	0.0049	0.0028	0.0008	0.0066	0.0059	0.0039	0.0163	0.0063	0.0065	0.0065	0.0052	0.0062	0.0015	0.0039	0.0048	0.0136	0.0132	0.0027	0.0041	0.0042	0.0080	0.0052	0.0002
Hainan	0.0004	0.0009	0.0032	0.0001	0.0025	0.0013	0.0009	0.0172	0.0053	0.0067	0.0012	0.0054	0.0020	0.0007	0.0011	0.0019	0.0131	0.0128	0.0013	0.0009	0.0008	0.0019	0.0015	0.0002
Chongqing	0.0018	0.0031	0.0034	0.0022	0.0042	0.0023	0.0016	0.0067	0.0078	0.0068	0.0025	0.0055	0.0044	0.0027	0.0044	0.0040	0.0160	0.0092	0.0070	0.0049	0.0082	0.0023	0.0028	0.0007
Sichuan	0.0036	0.0082	0.0029	0.0029	0.0105	0.0080	0.0018	0.0064	0.0087	0.0066	0.0094	0.0050	0.0137	0.0038	0.0083	0.0086	0.0155	0.0082	0.0067	0.0124	0.0068	0.0037	0.0076	0.0003
Guizhou	0.0013	0.0036	0.0024	0.0008	0.0066	0.0033	0.0012	0.0138	0.0075	0.0063	0.0017	0.0048	0.0064	0.0020	0.0029	0.0068	0.0153	0.0089	0.0016	0.0007	0.0009	0.0013	0.0019	0.0005
Yunnan	0.0017	0.0047	0.0026	0.0009	0.0069	0.0057	0.0022	0.0129	0.0083	0.0062	0.0047	0.0053	0.0090	0.0051	0.0039	0.0053	0.0145	0.0117	0.0027	0.0036	0.004	0.0058	0.0030	0.0008
Xizang(Tibet)	0.0001	0.0003	0.0022	0.0000	0.0008	0.0011	0.0027	0.0229	0.0060	0.0061	0.0014	0.0045	0.0002	0.0006	0.0003	0.0011	0.0110	0.0027	0.0001	0.0003	0.0022	0.0020	0.0017	0.0003
Shaanxi	0.0021	0.0038	0.0029	0.0019	0.0113	0.0081	0.0064	0.0083	0.0075	0.0066	0.0062	0.0052	0.0064	0.0034	0.0043	0.0076	0.0139	0.0095	0.0033	0.0055	0.0019	0.0059	0.0014	0.0122
Gansu	0.0007	0.0026	0.0023	0.0005	0.0043	0.0048	0.0051	0.0111	0.0058	0.0054	0.0051	0.0054	0.0050	0.0021	0.0019	0.0038	0.0144	0.0025	0.0025	0.0026	0.0057	0.0037	0.0021	0.0037
Qinghai	0.0003	0.0006	0.0027	0.0001	0.0018	0.0016	0.0020	0.0171	0.0065	0.0054	0.0019	0.0050	0.0026	0.0005	0.0006	0.0012	0.0136	0.0013	0.0008	0.0004	0.0031	0.0020	0.0012	0.0003
Ningxia	0.0003	0.0007	0.0029	0.0003	0.0009	0.0014	0.0012	0.0178	0.0089	0.0066	0.0013	0.0052	0.0019	0.0008	0.0007	0.0013	0.0110	0.0027	0.0022	0.0016	0.0043	0.0016	0.0012	0.0001
Xinjiang	0.0011	0.0024	0.0028	0.0003	0.0030	0.0038	0.0022	0.0186	0.0061	0.0064	0.0044	0.0049	0.0028	0.0018	0.0025	0.0042	0.0103	0.0010	0.0026	0.0042	0.0031	0.0072	0.0017	0.0003

Note: For the names and units of each index *SC*_1_ to *SC*_24_, please refer to the definitions of the sub-criteria in Table 1

**Table 7:** The results of the comprehensive evaluation of the sustainability of competitive sports in each region of China from 2013 to 2020.

	positive ideal solution	negative ideal solution	similarity	rank
Zhejiang	0.041575	0.0665046	0.61533	1
Jiangsu	0.042341	0.0626335	0.59665	2
Guangdong	0.050038	0.0570254	0.53263	3
Shandong	0.052986	0.0530777	0.50043	4
Henan	0.060496	0.0397035	0.39625	5
Hubei	0.062593	0.0408535	0.39492	6
Hebei	0.066223	0.0418284	0.38711	7
Beijing	0.070930	0.0379426	0.34851	8
Fujian	0.072021	0.0333796	0.31669	9
Hunan	0.071543	0.0321697	0.31018	10
Sichuan	0.072081	0.0323794	0.30997	11
Anhui	0.073642	0.0290281	0.28273	12
Jiangxi	0.074367	0.0280409	0.27382	13
Guangxi	0.075883	0.0278104	0.26820	14
Liaoning	0.074385	0.0271816	0.26762	15
Shaanxi	0.075100	0.0274048	0.26735	16
Yunnan	0.076279	0.0255213	0.25070	17
Shanghai	0.075450	0.0245536	0.24553	18
Xinjiang	0.081879	0.0232198	0.22093	19
Tianjin	0.089689	0.0251559	0.21904	20
Guizhou	0.082992	0.0221007	0.21030	21
Hainan	0.087633	0.0230449	0.20822	22
Shanxi	0.078249	0.0204259	0.20700	23
Heilongjiang	0.080717	0.0206375	0.20362	24
Inner Mongolia	0.079955	0.0196099	0.19696	25
Chongqing	0.082179	0.0201258	0.19672	26
Ningxia	0.088586	0.0210960	0.19234	27
Gansu	0.082102	0.0187112	0.18560	28
Qinghai	0.088507	0.0200149	0.18443	29
Jilin	0.083169	0.0162619	0.16355	30
Xizang(Tibet)	0.085796	0.0133097	0.13430	31

### Research result and discussion

This study will be discussed in two parts. The first part is the results of the overall development, which will examine the overall development of competitive sports in each region from 2013 to 2020. The second part presents a discussion of the development of competitive sports in the DPSIR (various criteria) in terms of each region, as detailed below.

#### The comprehensive development of competitive sports

Based on the calculation paradigm of 4.3 TOPSIS in this study, the similarity rankings of 31 provinces in China are as follows, with Zhejiang, Jiangsu, Guangdong, Shandong, and Henan ranking in the top five, and Ningxia, Gansu, Qinghai, Jilin, and Tibet ranking in the bottom five. The similarity ranking is a general reflection of the comprehensive socio-economic development level of each province, which fully demonstrates the sustainable development level of competitive sports in each province. The data for each year was disaggregated and the results were calculated in this study and the results obtained are shown in Table 8.

**Table 8:** Scores and rankings of sustainable development of competitive sports by region in China.

	2013	2014	2015	2016	2017	2018	2019	2020	overall score	rank
Zhejiang	0.66155	0.49224	0.62224	0.61126	0.61316	0.52157	0.60149	0.64476	0.63533	1
Jiangsu	0.58287	0.60001	0.57427	0.56369	0.54313	0.53626	0.61977	0.53900	0.59665	2
Guangdong	0.47980	0.44787	0.46405	0.50410	0.53210	0.53326	0.56341	0.58299	0.53263	3
Shandong	0.47920	0.51501	0.50763	0.52286	0.52286	0.43220	0.48329	0.45579	0.50043	4
Henan	0.34605	0.41220	0.37421	0.37331	0.37212	0.38201	0.41645	0.38729	0.39625	5
Hubei	0.30740	0.45248	0.33919	0.37039	0.36358	0.35288	0.44435	0.38679	0.39492	6
Hubei	0.31035	0.30155	0.29950	0.30068	0.29440	0.44002	0.34261	0.36538	0.38711	7
Hebei	0.38016	0.32448	0.36074	0.31935	0.30725	0.35542	0.36521	0.36295	0.34851	8
Beijing	0.27699	0.29094	0.30721	0.30166	0.30204	0.31977	0.35011	0.35513	0.31669	9
Fujian	0.28500	0.27941	0.29512	0.31813	0.30982	0.29343	0.33503	0.30223	0.31018	10
Hunan	0.33667	0.30332	0.30234	0.29761	0.29174	0.29059	0.33793	0.30695	0.30997	11
Anhui	0.25837	0.25928	0.27157	0.26871	0.26238	0.27896	0.33274	0.33965	0.28273	12
Jiangxi	0.31307	0.26470	0.27201	0.25024	0.24750	0.26274	0.29093	0.31958	0.27382	13
Guangxi	0.24826	0.29133	0.25231	0.25085	0.25161	0.27546	0.29326	0.34963	0.26820	14
Liaoning	0.30947	0.31455	0.27929	0.27229	0.25599	0.26728	0.26548	0.21096	0.26762	15
Shaanxi	0.25930	0.22871	0.24202	0.31332	0.22927	0.26950	0.29696	0.30762	0.26735	16
Yunnan	0.25986	0.23183	0.23765	0.23916	0.23779	0.24718	0.27588	0.29668	0.25070	17
Shanghai	0.27385	0.24134	0.22337	0.24921	0.25183	0.24607	0.27286	0.28069	0.24553	18
Xinjiang	0.24708	0.23171	0.25268	0.23374	0.22337	0.17625	0.14991	0.21236	0.22093	19
Tianjin	0.19378	0.18331	0.19534	0.18539	0.18138	0.21684	0.24528	0.39321	0.21904	20

The regional sustainable development level of competitive sports was estimated for each of the 31 provinces, municipalities and autonomous regions for eight consecutive years (2013–2020). As shown in Table 8, of the top 20 provinces, 13 provinces (65%) experienced an increase in the level of sustainable development, while 7 provinces (35%) experienced a decrease in the level of sustainable development. This indicates that the sustainable development of competitive sports in China as a whole has continued to improve, but individual provinces have shown little decline or small fluctuations. These include small fluctuations in provinces such as Zhejiang and Jiangsu, which are already at a high level of sustainable development, and low-level oscillations in provinces such as Xinjiang and Sichuan, which are relatively behind in terms of socio-economic development. Zhejiang, Jiangsu and Guangdong provinces ranked in the top three in terms of overall scores for eight years, especially Guangdong Province, which has maintained a steady upward development trend over the eight-year period from 2013 to 2020. The two provinces of Zhejiang and Jiangsu have slightly wavered but kept a fairly high level of sustainable development of competitive sports as a whole, as shown in Fig 3. Shanghai, Xinjiang and Tianjin ranked in the bottom three out of 20 provinces and cities. The reason for this is that although Shanghai and Tianjin are well developed economically, they have obvious shortcomings in the natural population growth rate, greening coverage rate and forest coverage rate, which make them ranked lower under the comprehensive evaluation of the indicator system, while in Xinjiang, the economic indexes such as GNP and per capita disposable income have significantly pulled down their rankings.

Data source: Due to the limitation of space, only the top 20 provinces, municipalities and autonomous regions are listed; Guizhou, Hainan, Shanxi, Heilongjiang and Tibet are not on the list.

**Fig 2:**
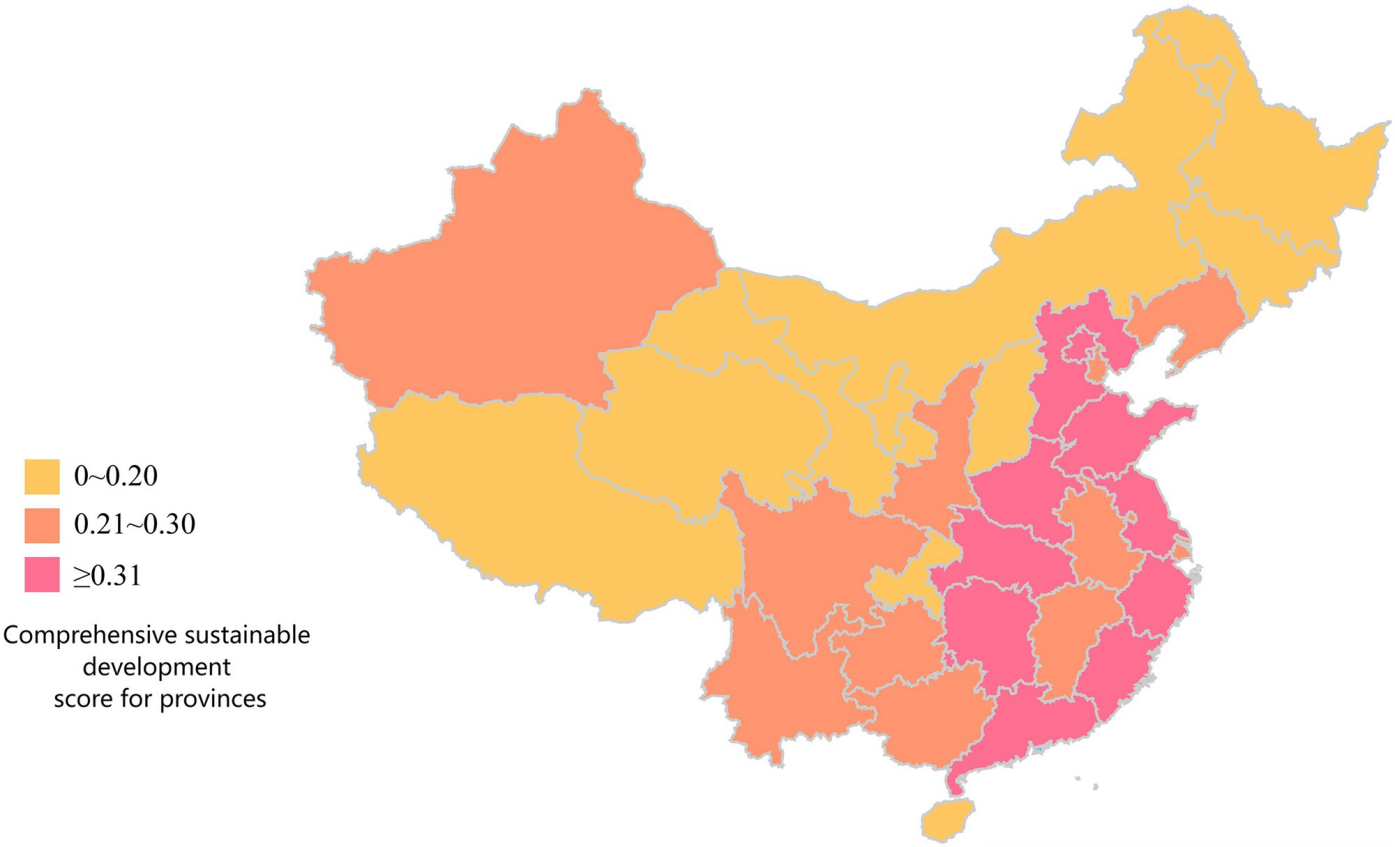
The cluster distribution of the average level of regional sustainable development of competitive sports in China [50]. Note: The online version is a color chart.

**Fig 3:**
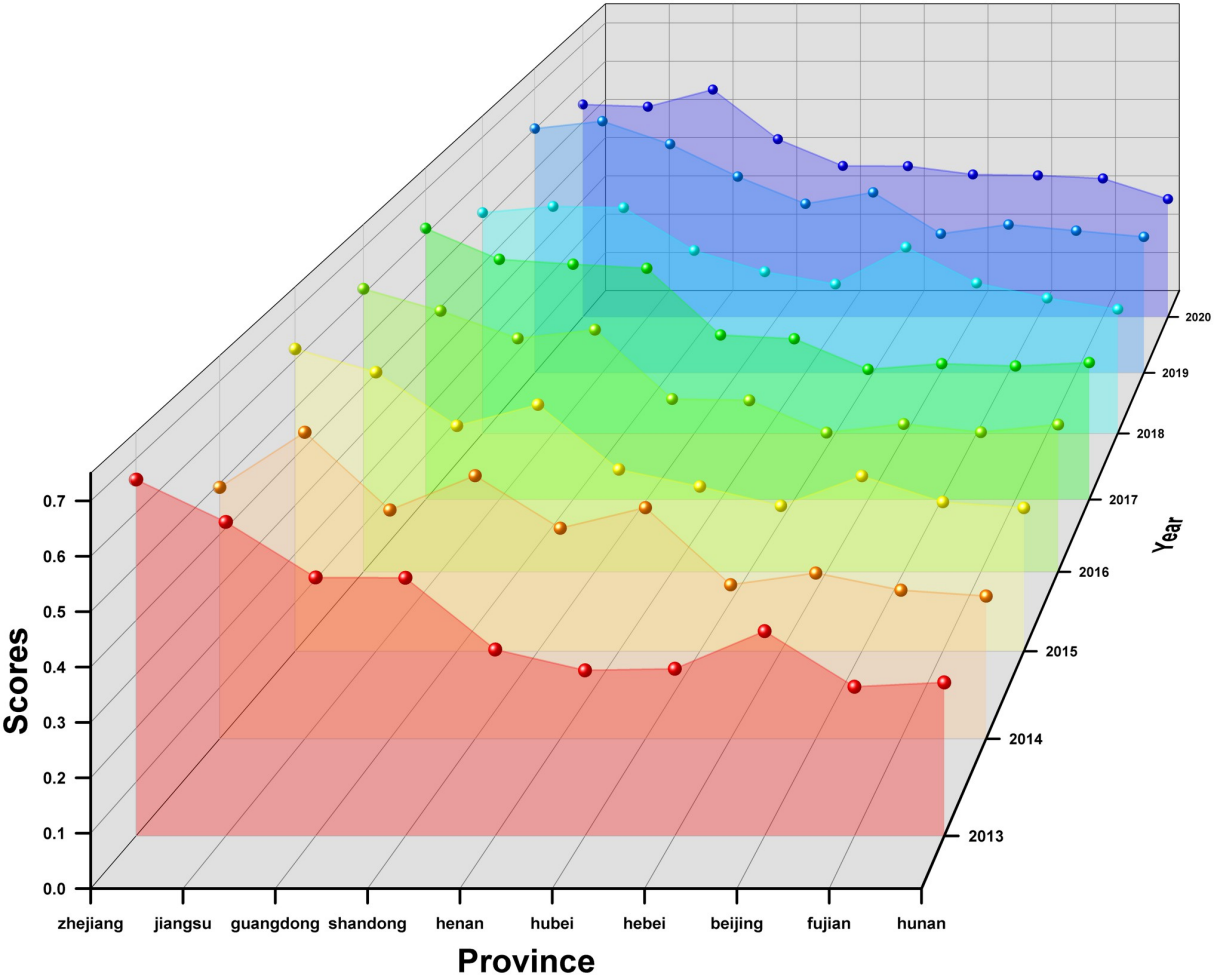
The 3-D trend of sustainable development of competitive sports in China’s provincial areas (top ten).

By using ArcGIS 10.7 to visualize the data from the 31 provinces in space (see Fig 2) and Table 9, the regional sustainability level of competitive sports in China is classified into three levels. The first level, with a sustainability score of ≥0.31, is primarily located in the eastern and southeastern provinces of China, including Zhejiang, Jiangsu, Guangdong, Shandong, Henan and ten other provinces (see Fig 3). The second level has a sustainability score between 0.21 and 0.31, mainly in Xinjiang and the central provinces of China, including eleven provinces, including Sichuan, Anhui, Jiangxi, Guangxi, Liaoning, Shaanxi, Yunnan, etc. The third level, with a sustainability score between 0 and 0.20, is mostly in the northwest as well as the northeast provinces of China, and includes ten provinces such as Hainan, Shanxi, and Tibet. All in all, the level of sustainable development of competitive sports in China roughly reveals a significant improvement from the northwest to the southeast.

**Table 9:** The average score clustering of the level of sustainable development of competitive sports in each province.

Score	Level 1 (Sustainability score ≥ 0.31)	Level 2 (0.21 ≤ sustainability score ≤ 0.30)	Level 3 (0 < sustainability score ≤ 0.20)
Province	Zhejiang, Jiangsu, Guangdong, Shandong, Henan, Hubei, Hebei, Beijing, Fujian, Hunan	Sichuan, Anhui, Jiangxi, Guangxi, Liaoning, Shaanxi, Yunnan, Shanghai, Xinjiang, Tianjin, Guizhou	Hainan, Shanxi, Heilongjiang, Inner Mongolia, Chongqing, Ningxia, Gansu, Qinghai, Jilin, Tibet
Region	Mainly in the eastern and the south-eastern provinces	Mainly in Xinjiang and the central provinces	Mainly in the north-western and the north-eastern provinces

#### The assessment and analysis of various sub-systems of the level of sustainable development of the competitive sports in China

(1) Drive Subsystem

**Fig 4:**
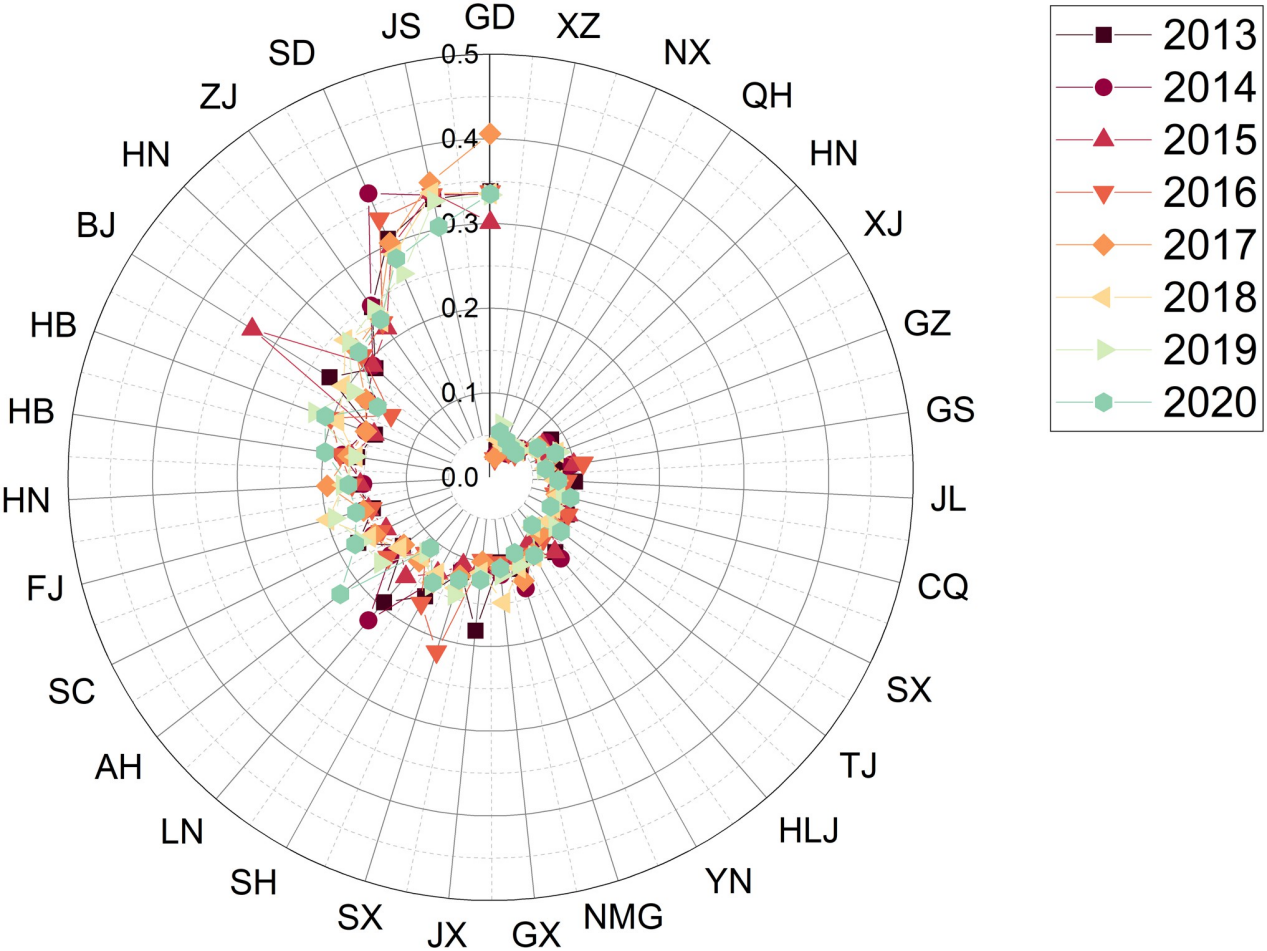
The score comparison of the subsystem of drivers of the sustainable development of competitive sports in each region of China.

It is shown in Fig 4 that during the 8-year period from 2013 to 2020, the provinces of Guangdong, Jiangsu, Shandong, Zhejiang, and Henan in China’s competitive sports sustainable development maintained a high average development level in the driver subsystem, while that of in the provinces of Tibet, Ningxia, Qinghai, Hainan, and Xinjiang was inferior to the average. This is closely related to the level of economic development of the province where it is located, and the better economic strength brings a stronger internal drive to sport economic. In addition to this, concerning the stability of the development of the driver subsystem, a certain degree of fluctuation has occurred in individual region, such as Shandong in 2014, Beijing in 2015, Jiangsu in 2017, Guangdong in 2017, Shaanxi in 2016, and Gansu in 2016 where there were large swings in the driver indexes. The overall view is that most of the provinces in China have shown a flat or slightly rising trend in terms of driving force, reflecting that China’s high-speed economic development has contributed to the driving force of the entire competitive sports, and reflecting the continuous emergence of China’s competitive sports’ ability to develop internally along with the high-quality development of the economy.

(2) Pressure Subsystem

**Fig 5:**
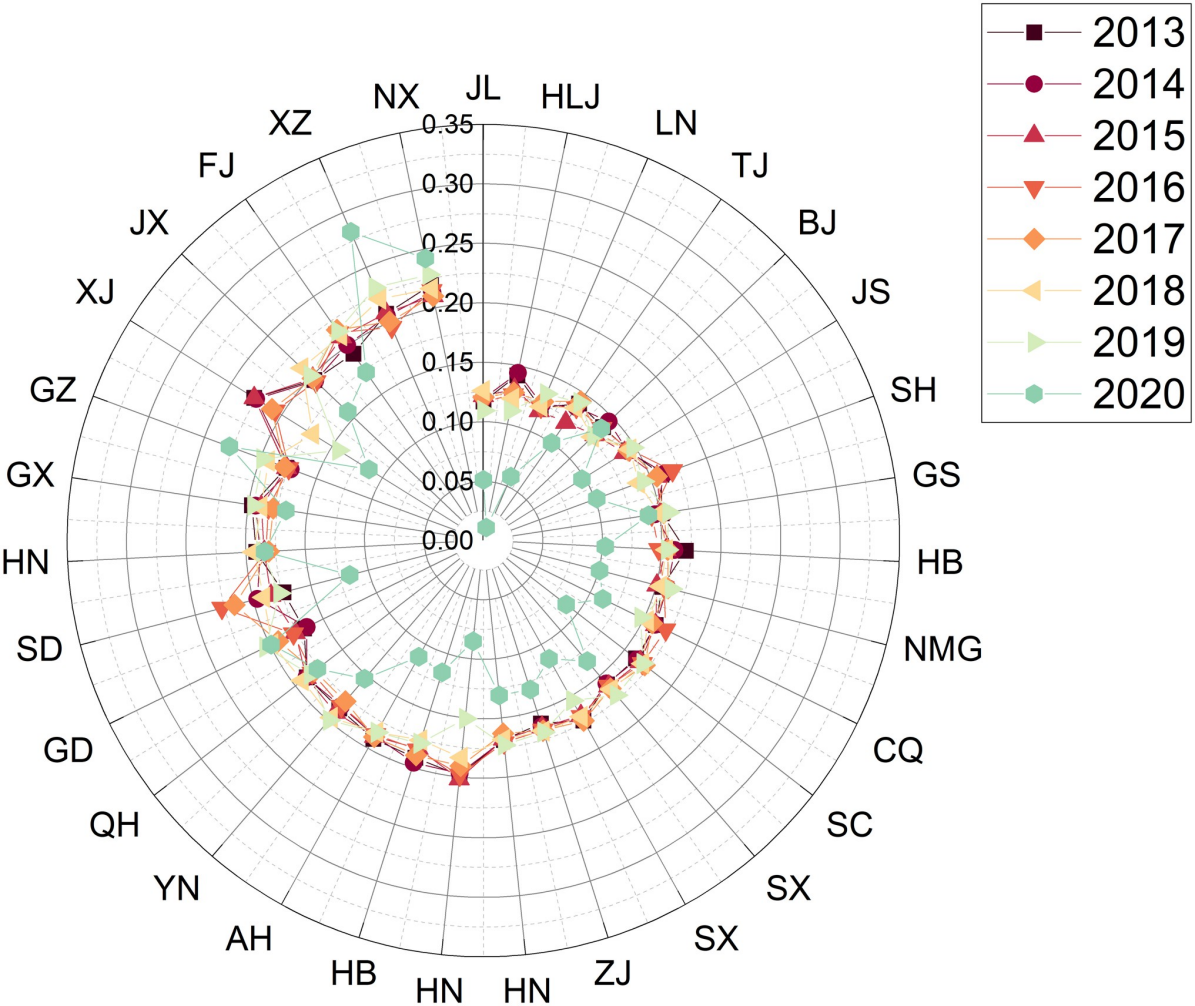
The comparison of the scores of the pressure subsystem for the sustainable Development of the competitive sports in each region of China.

As depicted in Fig 5, the provinces of Ningxia, Tibet, Fujian, Jiangxi, and Xinjiang maintained a high level in the pressure subsystem from 2013 to 2020. Conversely, the provinces of Beijing, Tianjin, Liaoning, Heilongjiang, and Jilin exhibited low levels of pressure. The reason for this is related to the fact that the pressure subsystem primarily responds to demographic and environmental factors. The five provinces at the top of the ranking showed a significant population growth rate and high green coverage rate. On the other hand, the five provinces at the bottom of the ranking, particularly Liaoning, Jilin, and Heilongjiang, have experienced a persistently low population growth rate. These provinces, known for heavy industry, face challenges in the form of resource over-exploitation and environmental degradation, which hinder the development of their competitive sports. The average pressure system level values of the 31 provinces over the 8-year period have shown a significant and consistent downward trend. As can be seen in Fig 5, the 2020 indexes of most provinces are lower than the average of the previous seven years. This is primarily attributed to the sharp decline in China’s birth rate in recent years. During these eight years, the average development level of China’s competitive sports reacting to the pressure subsystem showed a tendency to decline. The decrease in the birth rate of the population is the major reason for this, and this is a point that must be taken into account in the future development of competitive sports in China.

(3) State Subsystem

**Fig 6:**
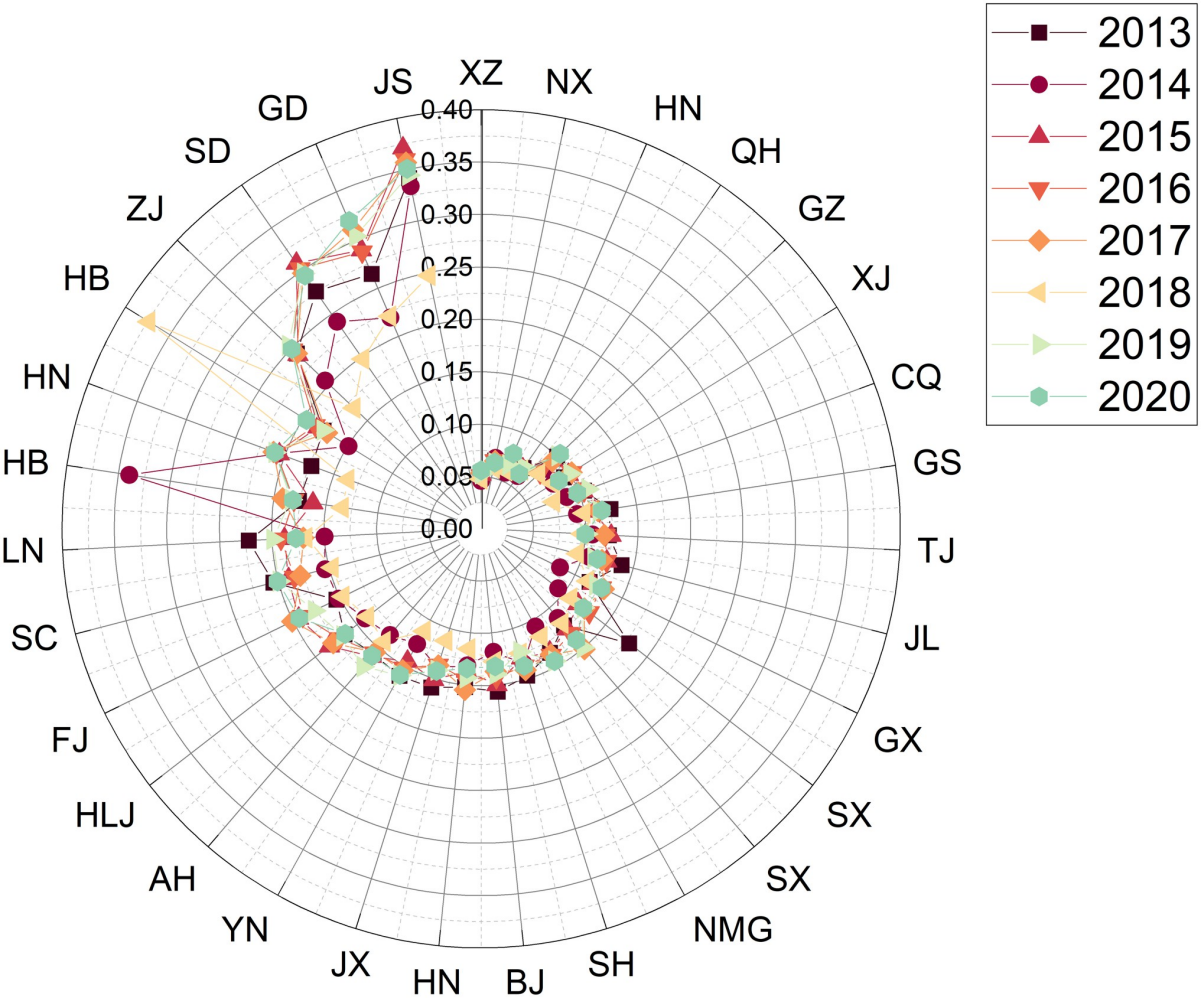
The comparison of the state subsystem scores of the sustainable development of the competitive sports in each region of China.

As shown in Fig 6, the five provinces of Jiangsu, Guangdong, Shandong, Zhejiang, and Hebei ranked in the top five of the state subsystems during the 8-year period, and Tibet, Ningxia, Hainan, Qinghai, and Guizhou ranked in the bottom five. The indexes related to the state subsystem, such as the number of athletes in outstanding sports teams and the sales of sports lottery tickets, are directly related to competitive sports. The huge gap between the top five provinces and the bottom five provinces can be observed very dramatically in this regard. While the state subsystems showed a relatively flat but gradually increasing level, the eastern and central provinces in particular showed a better development trend. However, the western provinces, especially Tibet, Ningxia, and Qinghai in the north-west, exhibited a tendency to grow increasingly distant from the central and eastern provinces. Jiangsu, which ranked first, had a composite score of 0.33812 for eight years, while Tibet, which ranked last, had a composite score of 0.05100 for eight years, thus showing the huge gap between them. Furthermore, while Jiangsu has consistently achieved a high score for eight years, Tibet has remained at a low level, showing minimal growth in 2020 compared to 2013, which fully revealed the imbalance in the distribution of resources for competitive sports in China. This is what needs to be emphasized and changed for the future high-quality development of competitive sports in China.

(4) Impact subsystem

**Fig 7:**
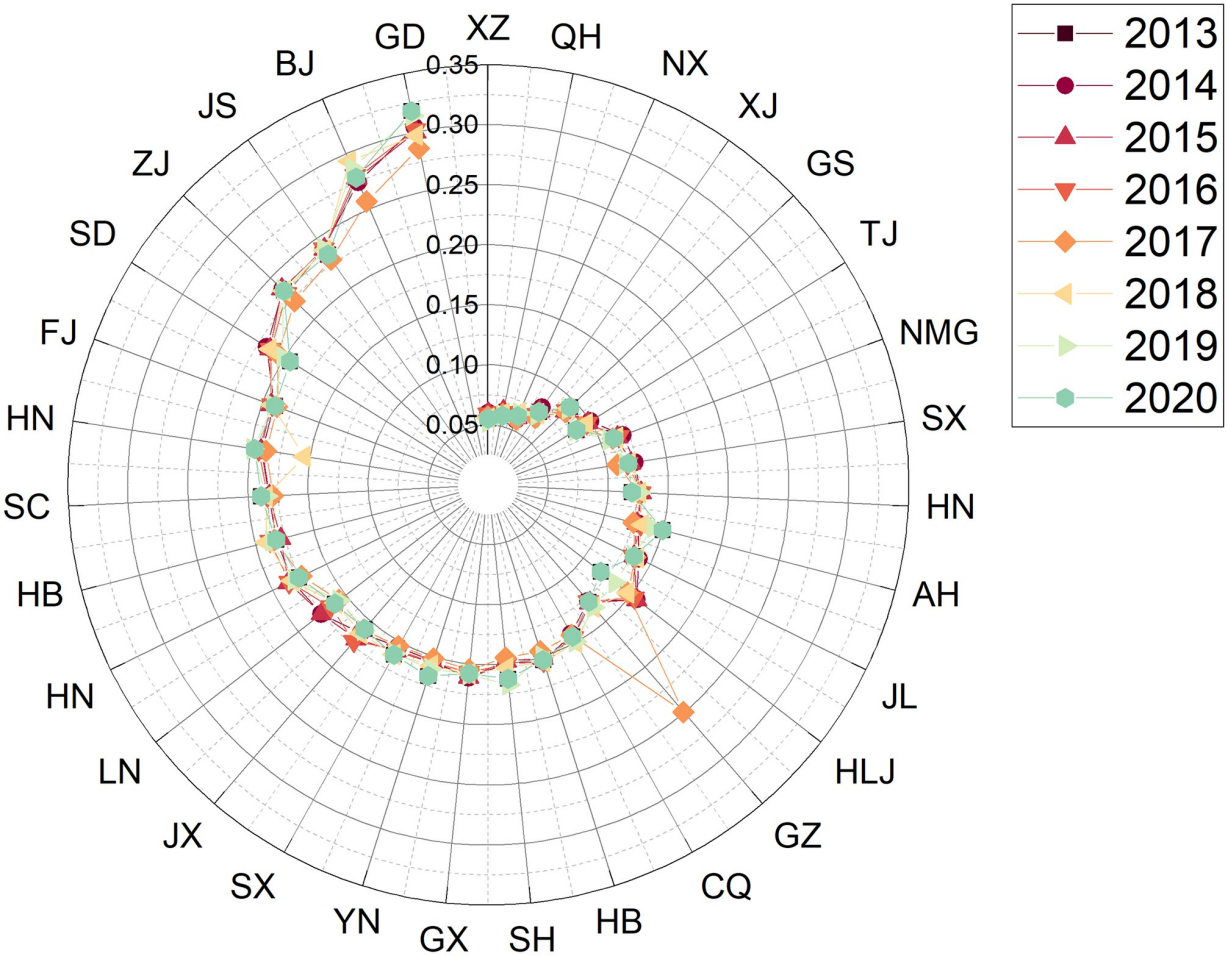
The comparison of the impact subsystem scores of the sustainable development of the competitive sports in different regions of China.

Over the eight-year period from 2013 to 2020, the provinces of Guangdong, Beijing, Jiangsu, Zhejiang, and Shandong consistently ranked in the top five in terms of impact subsystems. Conversely, Gansu, Xinjiang, Ningxia, Qinghai, and Tibet provinces consistently ranked in the bottom five, as depicted in Fig 7. The impact subsystem primarily reflects the province’s integrated economic, social, and natural attributes, with the southeastern provinces remaining overwhelmingly superior, and there is a huge socio-economic disparity between the northwestern and southeastern regions. Even if some western provinces have a slight advantage in the index of “the forest coverage rate”, it is far from sufficient to compensate for it. Compared to the eastern and central provinces, the impact level values of the next five provinces also remained almost unchanged between 2013 and 2020. This reflects the sluggish development of the impact subsystem of competitive sports in the Western region. In summary, the development level of the impact subsystem in most provinces revealed a steady growth with a slight increase. This indicates that the impact system in most provinces is gradually improving with minor fluctuations. However, the gap between the eastern and western provinces has not been narrowed over time, which is a significant issue that needs to be addressed.

(5) Response subsystem

**Fig 8:**
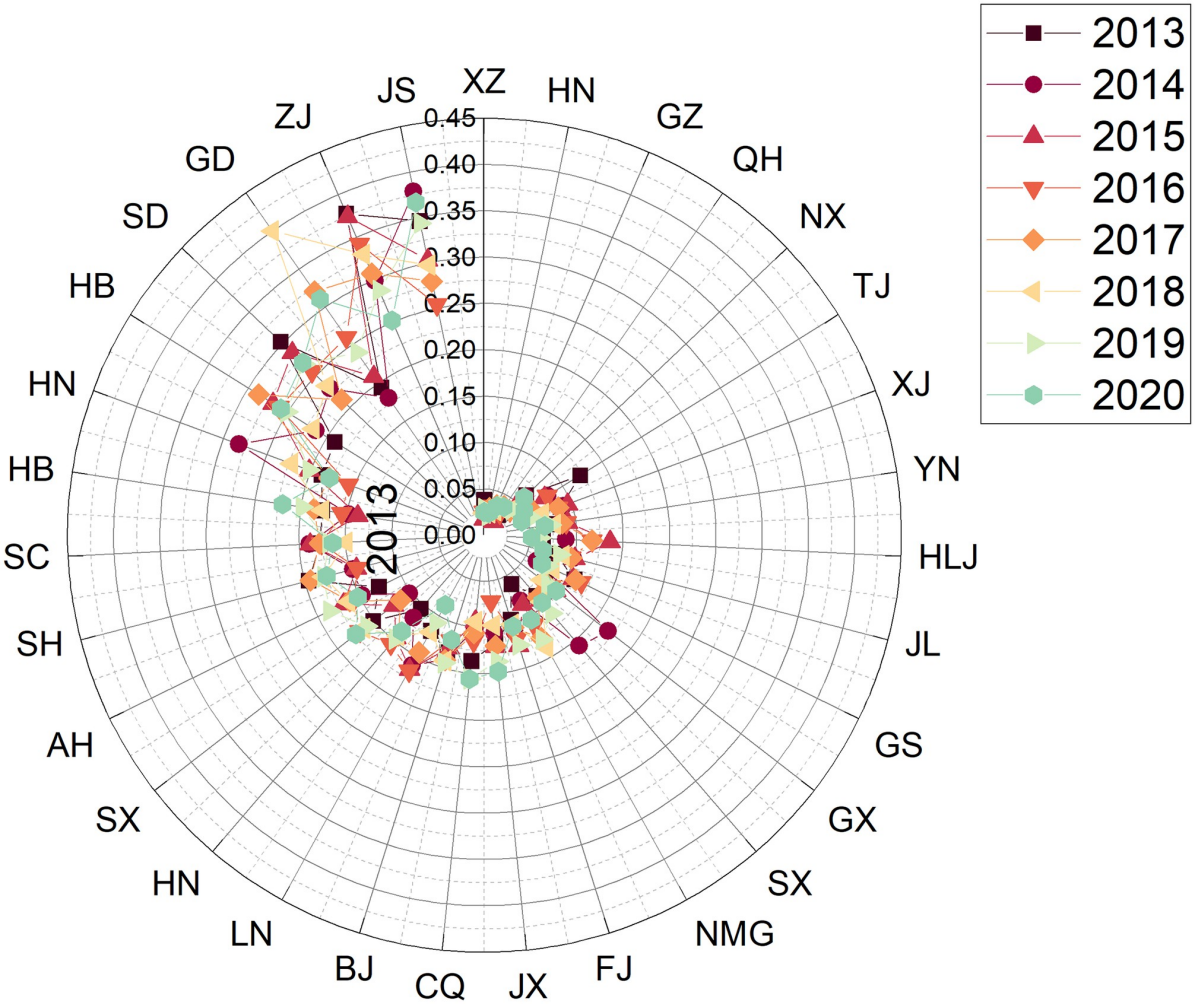
The comparison of the impact subsystem scores of the sustainable development of the competitive sports in different regions of China.

Over the eight years of 2013–2020, as shown in Fig 8, Jiangsu, Zhejiang, Guangdong, Shandong, and Hubei provinces ranked in the top five of the response subsystem scores, and Ningxia, Qinghai, Guizhou, Hainan, and Tibet ranked in the bottom five. The response subsystem mainly manifests the degree of response of each province under the influence of the driver subsystem and the pressure subsystem, and the six indexes related to this subsystem are the outcome indexes that fully reflect the potential of the development of competitive sports in the provincial area. The enormous influence of economic strength on the core index of a province’s competitive sport remains very evident in this subsystem score. Jiangsu, Zhejiang and Guangdong are without exception the leading provinces in China’s economic development, while Ningxia, Qinghai and Guizhou are also unsurprisingly the more backward provinces, thus demonstrating the important role of a province’s economic base. As a whole, the development of the subsystem is more volatile, with the subsystem score of Zhejiang Province dropping from 0.37765 to 0.252229 from 2013 to 2020, representing a drop of 33.21%, while the subsystem score of Guangdong Province rose from 0.19396 to 0.310166, representing an increase of 59.91%. Besides, the majority of the provinces’ scores on this subsystem index fluctuate considerably because it is strongly influenced by the other four subsystems. This subsystem is subject to variations in the other four subsystems and therefore exhibited greater volatility in the system score. To sum up, the eastern and the central regions obtained higher scores in the response subsystem, the north-western region obtained lower scores, most of the provinces showed some volatility, and the gap between the eastern and western regions was larger, and half of the provinces had a decreasing trend. Despite the fact that each province has taken certain measures, the effect is not desirable, and it is necessary to further improve the effectiveness of the governance of the competitive sports in each province and to put the policy into practice.

## Conclusion and suggestion

### Conclusion

The main contribution of this paper lies in addressing the key problems in the field of competitive sports in China, proposing solutions, and constructing a model for the comprehensive evaluation of the sustainable development level of competitive sports in the provinces. China’s competitive sports face challenges such as unbalanced regional development [2], organizational conflicts [5], low market participation, delayed professional reform, and low technological capability, which have become obstacles to China’s efforts to build a sports powerhouse. Based on the “Evaluation Indicator System for the Sustainable Development of Chinese Athletics”, this paper conducted an in-depth evaluation of the sustainable development level of athletics in 31 provinces, autonomous regions and municipalities directly under the Central Government of China by using the “DPSIR-TOPSIS” model and the entropy weighting method. The sustainable development level of athletics in 31 provinces, autonomous regions and municipalities directly under the Central Government of China has been thoroughly evaluated. So far, there has been no in-depth study on the sustainable development of provincial competitive sports in the Chinese context, and this study fills this gap. Through the constructed evaluation model and methodology, this paper not only provides a comprehensive assessment of the level of sustainable development of competitive sports, but also provides a set of systematic and scientific decision-making reference bases for government departments or relevant decision-making organizations. The main findings of this paper are summarized below.

(1) Provincially: Jiangsu, Zhejiang and Guangdong lead in both the overall level of the sustainable development and the rankings of the four subsystems (except for the pressure subsystem), while Tibet, Qinghai and Ningxia are in the most backward position, and the central provinces are in the middle of the pack. The central provinces of Shandong, Henan and Hubei, on the other hand, shone in the sustainable development level of the competitive sports.
(2) Spatially: Whether in the ranking of the overall level of sustainable development or in the ranking of the five subsystems, it basically presents the state of East > Centre > West. In addition to the pressure subsystem, which presented the phenomenon of West > Centre > East due to its unique index setting, the eastern provinces demonstrate greater sustainability in terms of their capacity for competitive sports development. The level of sustainable development in competitive sports is influenced by significant geographical advantages and regional characteristics.
(3) Timing: From 2013 to 2020, the level of sustainable development in competitive sports has shown an upward trend in most provinces, with only a few provinces experiencing a slight decline or small fluctuations. Generally speaking, the level of sustainable development is favorable, with provinces that have better development momentum further distancing themselves from the less favorable provinces. However, there is no obvious trend for the less favorable provinces, particularly in the central and western parts of the country, to catch up with each other.

### Suggestion

(1) Implementation of a balanced development strategy for competitive sports in provincial areas

With the result of the evaluation of the overall level of sustainability of competitive sports, it is recommended that the government continue to strengthen the support for the development of competitive sports in the leading provinces of Jiangsu, Zhejiang and Guangdong to ensure their leading position in sustainability. For provinces that are relatively backward, such as Tibet, Qinghai and Ningxia, the government can put forward special policies and programs to improve the sustainable development of competitive sports and narrow the gap with the leading provinces. It is also necessary to understand the differences in the level of sustainable development of competitive sports in the eastern, central and western regions and to formulate regionally differentiated development strategies based on the results of the study. Particular attention should be paid to the establishment of indicators for the stress subsystem in order to understand the specific situation of the Western Region and to take appropriate measures to improve the sustainability of competitive sport in the region. The government will also need to continue to monitor time trends in the level of sustainable development of competitive sports, paying special attention to provinces that have experienced declines or small fluctuations. A long-term development plan should be formulated to ensure that the level of sustainable development of competitive sports continues to improve, especially in the central and western provinces. Encourage the exchange of experience and cooperation between leading provinces and relatively backward provinces to promote the improvement of the level of sustainable development of competitive sports. Central provinces should be supported in their good performance and encouraged to further develop competitive sports and pay more attention to policy and resource allocation.

(2) Constructing a new type of national sports organisational system with effective governmental and market-oriented measures.

The omnipotent government’s extensive allocation of resources for competitive sports has contributed to the remarkable achievements in China’s history. Yet, under the new pattern of competition in the world’s competitive sports, the unidirectional hierarchical management system of the government has become increasingly inadequate to adapt to the new development trend of global competitive sports. The new national system should not rely solely on the strength of the government but should mobilize the strength of individuals, families, society, and other diverse entities. The report of the 20th National Congress of the Communist Party of China pointed out that, “while giving full effect to the decisive role of the market in the allocation of resources and giving better play to the role of the government, it is necessary to form a governance pattern in which the government and the market are organically integrated, complementary, co-ordinated and mutually reinforcing.” The synergy of individuals, families, society, and other multiple subjects is particularly important in this process, taking full advantage of multiple subjects in terms of coordination of interests, high efficiency, and flexibility, as well as competition and transparency, to form a new framework for the governance of the competitive sports that unites a competent government and an effective market. The modernization of the competitive sports governance system can be achieved if the comparative advantages of the 31 provinces, municipalities, and autonomous regions with respect to their economic aggregates, natural resources, and social organization can be brought into play to form a synergistic, efficient and open competitive sports governance system.

(3) Promoting the integration of the competitive sports.

For a long time, the social function of competitive sports in China has not been fully explored. Standing at the threshold of the 14th Five-Year Plan, competitive sports should align with China’s national strategy, political interests, and political demands in the new era. To the international community, sports serve as a bridge to promote the new era of great power diplomacy with Chinese characteristics, optimising the geopolitical environment and showcasing China’s image as a peacefully rising great power. Domestically, competitive sports play a crucial role in promoting social development and fostering social integration. They also contribute to maintaining equality, harmony, stability, and national unity. In the economic sphere, competitive sports fully utilize the potential economic value of sports. They support systematic and comprehensive policies for professional sports, leisure sports, sports tourism, and other related sectors. This helps meet the increasing demands of people for a better quality of life and contributes to supply-side reform. The development of competitive sports should focus on broader political and economic goals so that it can better serve the rapid development of the country’s political and economic sectors. This will enable competitive sports to become a visible indicator of the great rejuvenation of the Chinese nation.

(4) Enhancing the innovation-driven capacity of the provincial competitive sport.

In the new era, China’s competitive sports have transitioned from the stage of reform and development to the stage of high-quality development. The drive for innovation is an important source of inspiration for maintaining the high quality of provincial competitive sports in the future. It is also important for the 31 provinces to meet the challenge of staying on track in the new stage of development. For instance, the innovation of talent selection and training mode, represented by “the cross-border cross-discipline selection,” made a significant contribution to winning gold and silver in the Beijing Winter Olympic Games. In this regard, the 31 provinces must embark on an innovation drive to enhance the mechanism for the high-quality development of competitive sports. This includes implementing the top-level design for sport’s reform, promoting collaborative governance among diverse stakeholders, adopting a diversified talent cultivation model, implementing scientific and intelligent training methods, developing effective strategies for preparing for large-scale games, integrating sports and education, creating incentive models for competitive athletes, and fostering cultural creativity in the competitive sports program, etc. Evidently, this innovation drive will greatly contribute to the sustainable development of competitive sports in the province.

(5) The implementation of the strategy for the development of the comparative advantages of competitive sports in the provincial areas.

The 31 provinces should integrate their own comparative advantages and resources as well as the basic conditions of the provinces, search for the development approach that suits them, plan the layout of provincial competitive sports scientifically, and explore the development model of the provincial competitive sports with high-quality development. For the top-ranking provinces such as Zhejiang, Jiangsu, Shandong and other provinces with better sustainable development indexes of competitive sports, the layout of program should be further refined, the industrial structure of competitive sports should be enriched, and the comprehensive integration of competitive sports and social life should be further intensified. The provinces in the central and western parts of China with poor indexes of sustainable development of competitive sports should enhance the key program suitable for the resource endowment of the province on the basis of making up for the short boards, so as to bring the whole area to the forefront and lead the way with the advantageous program to continuously narrow the gap with the eastern provinces, and to push forward the high-quality sustainable development of competitive sports in the country.

### Shortcomings and future perspectives of this study

In this paper, Entropy and TOPSIS were used only for the analysis and other methods of MCDM were not considered for the methodological use, such as Complex Proportional Assessment (COPRAS), Weighted Aggregates Sum Product Assessment (WASPAS). Simultaneous Evaluation of Criteria and Alternatives (SECA), Combinative Distance-based Assessment (CODAS), Stepwise Weight Assessment Ratio Analysis II (SWARA II), MEthod based on the Removal Effects of Criteria (MEREC) and Evaluation based on Distance from Average Solution (EDAS).

The limitations of the methodological choices can be summarized as follows.

(1) High data requirements: Entropy and TOPSIS require sufficient data for weighting calculations and evaluating alternatives. In the absence of data, the effectiveness of the method may be compromised.
(2) Subjective sensitivity to weights: Entropy weight calculations can be influenced by subjective judgments, which can lead to subjective results.
(3) Only considers relative similarity: TOPSIS mainly considers the similarity of alternatives relative to the ideal and anti-ideal solutions, which may ignore other important information.
(4) Inability to handle non-linear relationships: Entropy and TOPSIS may not be able to effectively handle situations where there are non-linear relationships between criteria.

As a result, different approaches have different theoretical underpinnings. For example, COPRAS uses complex proportionality assessment, WASPAS combines weighted sums and products, SECA allows simultaneous evaluation of criteria and alternatives, CODAS is based on combinatorial distances, SWARA II performs stepwise weighting assessment, MEREC is based on criterion removal effects, and EDAS is based on distances to the mean solution. Each method has a unique computational approach, including different treatments of criterion weights, alternatives assessment, and ranking, and these methods have different emphases and computational approaches in responding to multi-criteria decision problems, and the choice of which method to use should depend on the characteristics of the problem, the state of the information, and the needs of the decision-maker. At the same time, there may be potential application scenarios where these methods complement each other. Therefore, this paper proposes recommendations for future research to address the limitations described below.

(1) Broadening the scope of application: For each method, future research could focus on practical applications in different fields and application scenarios. This will help to assess the generalizability and adaptability of the methods.
(2) Uncertainty handling: Consider introducing uncertainty factors into these methods to better handle real-world uncertainty. This may include fuzzy logic, random factors, or other forms of uncertainty handling.
(3) Performance evaluation: Perform systematic performance evaluation to compare the performance of these methods in different contexts. This may include assessments of accuracy, computational efficiency, scalability, etc.
(4) Method Integration: Consider integrating the methods to improve the performance of the overall MCDM model. Possible integration approaches include using them in tandem, using them in parallel, or developing new integration frameworks.
(5) Handling subjectivity: Find ways to reduce the impact of subjectivity in the methods to improve their objectivity and verifiability.
(6) Multi-Objective Problems: Consider the application of these methods to multi-objective problems and examine their effectiveness in dealing with multi-criteria situations.

As a conclusion, this paper suggests that through in-depth research on these commonalities, these MCDM methods can be made to better cope with complex decision-making situations encountered, and their practical application value can be strengthened.

## Supporting information

S1 Data (ZIP)
