# Supplementary material for: Constructing an evaluation model for the comprehensive level of sustainable development of provincial competitive sports in China based on DPSIR and MCDM
Source: PLoS One. 2024 Apr 16;19(4):e0301411. doi: 10.1371/journal.pone.0301411 (PMC11020774; doi:10.1371/journal.pone.0301411)
Supplement: S1 Data — (ZIP) [file pone.0301411.s001.zip › Supporting Information files/Table 1. The evaluation index system for the sustainable development of competitive sports in China.docx]

**Table 1.** The evaluation index system for the sustainable development of competitive sports in China

| Criteria | Sub-criteria | Unit | Attributes |
| --- | --- | --- | --- |
| *C*_1_: Drive  (D) | *SC*_1_: Gross domestic product (GDP) | Billions of yuan | positive |
|  | *SC*_2_: Total population at year-end | 10,000 people | positive |
|  | *SC*_3_: Per capita disposable income of the whole population | yuan | positive |
|  | *SC*_4_: Research and development full-time equivalent staff | man-year | positive |
|  | *SC*_5_: The investment in fixed assets in culture, sports and recreation | Billions of yuan | positive |
|  | *SC*_6_: The number of employee in the sports system | people | positive |
|  | *SC*_7_: The number of fitness activity centers | number | positive |
| *C*_2_: Pressure  (P) | *SC*_8_: Crude birth rate  crude birth rate | % | positive |
|  | *SC*_9_: Unemployment rate of the urban population | % | negative |
|  | *SC*_10_: The Green Coverage Rate in Developed Areas | % | positive |
| *C*_3_: State  (S) | *SC*_11_: The number of the outstanding athletes of the sports teams | people | positive |
|  | *SC*_12_: The national physique qualified rate | % | positive |
|  | *SC*_13_: The number of the sports social organization | number | positive |
|  | *SC*_14_: sports lottery sales | 10,000 yuan | positive |
| *C*_4_: Impact  (I) | *SC*_15_: The growth value of the tertiary industry | Billions of yuan | positive |
|  | *SC*_16_: The Employment in culture, sports and recreation | 10,000 people | positive |
|  | *SC*_17_: Mortality rate | % | negative |
|  | *SC*_18_: forest coverage rate | % | positive |
| *C*_5_: Response  (R) | *SC*_19_:The number of the level athletes developed | people | positive |
|  | *SC*_20_: The number of the athletic reserves | people | positive |
|  | *SC*_21_: The number of the youth sports clubs | number | positive |
|  | *SC*_22_: The number of National Physical Fitness Monitoring Stations | number | positive |
|  | *SC*_23_: The number of National Fitness Trail Projects | number | positive |
|  | *SC*_24_: The number of sports research projects | number | positive |
