# Supplementary material for: Constructing an evaluation model for the comprehensive level of sustainable development of provincial competitive sports in China based on DPSIR and MCDM
Source: PLoS One. 2024 Apr 16;19(4):e0301411. doi: 10.1371/journal.pone.0301411 (PMC11020774; doi:10.1371/journal.pone.0301411)
Supplement: S1 Data — (ZIP) [file pone.0301411.s001.zip › Supporting Information files/Table 5.The statistical values of the indexes from 20013 to 2020 by regions in China.docx]

**Table 5.** The statistical values of the indexes from 20013 to 2020 by regions in China

| Criteria  Regions | *C*_1_: Drive（D） | | | | | | | *C*_2_: Pressure (P) | | | *C*_3_: State (S) | | | | *C*_4_: Impact (I) | | | | | *C*_5_: Response (R) | | | | | |
| --- | --- | --- | --- | --- | --- | --- | --- | --- | --- | --- | --- | --- | --- | --- | --- | --- | --- | --- | --- | --- | --- | --- | --- | --- | --- |
|  | *SC*_1_ | *SC*_2_ | *SC*_3_ | *SC*_4_ | *SC*_5_ | *SC*_6_ | *SC*_7_ | *SC*_8_ | *SC*_9_ | *SC*_10_ | *SC*_11_ | *SC*_12_ | *SC*_13_ | *SC*_14_ | *SC*_15_ | *SC*_16_ | *SC*_17_ | *SC*_18_ | *SC*_19_ | | *SC*_20_ | *SC*_21_ | *SC*_22_ | *SC*_23_ | *SC*_24_ |
| Shanghai | 29830.09 | 2442.25 | 57144.56 | 90661.25 | 88.64 | 4846.13 | 17.50 | 2.21 | 3.86 | 37.91 | 942.88 | 97.1 | 793.75 | 406101.67 | 20687.57 | 6.35 | 5.29 | 11.98 | 1819.13 | | 10850.25 | 391.75 | 247.88 | 1008.88 | 91.00 |
| Beijing | 51011.81 | 2168.96 | 55385.90 | 50971.75 | 146.20 | 5426.88 | 88.13 | 3.30 | 1.50 | 48.42 | 1046.88 | 91.6 | 577.63 | 632370.46 | 22181.72 | 18.50 | 5.17 | 38.81 | 1764.88 | | 23544.25 | 180.75 | 286.63 | 976.75 | 20.25 |
| Tianjin | 16263.66 | 1476.22 | 35417.96 | 63986.13 | 69.40 | 2794.25 | 15.25 | 1.35 | 3.53 | 36.66 | 812.25 | 93.0 | 349.50 | 333256.94 | 9148.90 | 2.23 | 5.65 | 10.70 | 1853.88 | | 5528.50 | 66.63 | 62.13 | 897.00 | 6.63 |
| Hebei | 32830.29 | 7433.53 | 20926.30 | 76642.25 | 499.04 | 6049.88 | 22.38 | 5.06 | 3.52 | 41.71 | 999.38 | 84.1 | 1685.63 | 3455191.97 | 14329.73 | 5.28 | 6.49 | 25.25 | 2217.75 | | 16116.25 | 265.75 | 186.38 | 3628.13 | 51.75 |
| Shanxi | 14690.83 | 3601.87 | 20001.61 | 30898.25 | 125.83 | 4472.88 | 9.25 | 4.04 | 3.28 | 41.10 | 563.50 | 93.0 | 1655.88 | 224958.88 | 7431.53 | 4.58 | 5.82 | 18.95 | 1582.00 | | 8771.75 | 103.25 | 198.88 | 1529.38 | 27.50 |
| Inner Mongolia | 17378.85 | 2475.32 | 25291.11 | 23223.50 | 141.10 | 3560.50 | 46.75 | 2.51 | 3.67 | 39.61 | 734.88 | 85.9 | 1595.63 | 368166.34 | 7747.83 | 3.41 | 5.91 | 21.43 | 1455.00 | | 12705.63 | 272.63 | 96.75 | 905.88 | 26.75 |
| Liaoning | 25737.74 | 4331.56 | 27043.53 | 54436.63 | 108.44 | 6909.75 | 45.13 | -0.85 | 3.81 | 40.01 | 1820.00 | 89.7 | 1352.25 | 435777.84 | 12378.57 | 4.88 | 7.02 | 38.62 | 1843.63 | | 15025.63 | 327.75 | 334.38 | 1285.25 | 4.75 |
| Jilin | 13718.35 | 2629.63 | 20831.23 | 18826.25 | 116.30 | 4355.13 | 10.75 | -0.33 | 3.46 | 36.41 | 639.13 | 87.8 | 1092.63 | 319040.58 | 5612.81 | 3.54 | 6.27 | 40.68 | 950.38 | | 9090.88 | 236.13 | 117.25 | 750.25 | 32.25 |
| Heilongjiang | 14970.60 | 3602.61 | 20603.28 | 25658.50 | 101.35 | 4966.63 | 17.13 | -0.83 | 4.09 | 36.00 | 1340.13 | 87.8 | 1439.38 | 410652.33 | 7582.67 | 3.81 | 6.81 | 43.39 | 1016.63 | | 9645.38 | 104.00 | 151.13 | 1794.38 | 14.50 |
| Jiangsu | 81412.43 | 8161.91 | 33933.43 | 458518.75 | 537.29 | 9149.25 | 72.63 | 1.94 | 3.03 | 42.96 | 1589.88 | 91.2 | 5576.25 | 1663645.99 | 40654.09 | 8.23 | 7.00 | 15.58 | 2475.13 | | 20868.13 | 544.38 | 824.25 | 4707.63 | 146.25 |
| Zhejiang | 50255.34 | 5863.50 | 40835.13 | 356550.13 | 350.42 | 5481.38 | 26.50 | 4.76 | 2.80 | 41.00 | 1069.88 | 93.7 | 3285.38 | 1093488.09 | 26214.97 | 7.33 | 5.57 | 59.21 | 2613.00 | | 21363.13 | 617.75 | 1645.88 | 3695.63 | 15.38 |
| Anhui | 27416.41 | 6122.48 | 21334.46 | 106543.75 | 230.33 | 4192.88 | 36.50 | 6.06 | 3.00 | 41.68 | 719.50 | 92.5 | 2142.50 | 495471.38 | 11940.27 | 3.44 | 6.22 | 27.95 | 1537.13 | | 16249.13 | 489.88 | 108.13 | 2060.00 | 50.38 |
| Fujian | 31839.45 | 3950.75 | 29133.91 | 113214.63 | 465.23 | 4968.63 | 28.75 | 6.80 | 3.69 | 43.50 | 1116.00 | 88.3 | 2047.13 | 635633.31 | 13940.19 | 4.23 | 6.16 | 66.27 | 1435.13 | | 23403.00 | 69.13 | 114.00 | 1277.88 | 21.75 |
| Jiangxi | 19798.45 | 4549.00 | 21346.34 | 52818.50 | 225.96 | 3621.50 | 32.50 | 6.43 | 3.26 | 45.05 | 570.25 | 91.6 | 1834.00 | 428744.07 | 8455.41 | 3.33 | 6.30 | 60.83 | 1293.25 | | 15702.63 | 159.63 | 218.50 | 911.13 | 41.13 |
| Shandong | 67310.27 | 9958.76 | 25984.73 | 233816.25 | 808.50 | 10790.63 | 52.13 | 6.20 | 3.33 | 42.16 | 1332.75 | 91.5 | 3976.75 | 1412876.65 | 32264.08 | 7.08 | 7.05 | 16.93 | 4165.88 | | 26317.88 | 171.00 | 412.63 | 4058.75 | 86.63 |
| Henan | 43320.72 | 9640.75 | 19539.15 | 132578.38 | 614.47 | 6499.13 | 29.63 | 4.85 | 3.03 | 39.40 | 552.50 | 91.5 | 2947.00 | 1044778.88 | 18570.25 | 7.51 | 6.99 | 22.49 | 2525.50 | | 13847.88 | 386.13 | 392.63 | 2540.38 | 100.88 |
| Hubei | 34750.01 | 5855.31 | 22792.51 | 100065.75 | 678.93 | 6159.00 | 29.63 | 4.17 | 2.83 | 38.49 | 2315.75 | 94.2 | 1749.75 | 624152.22 | 16043.84 | 6.25 | 6.86 | 38.85 | 2272.00 | | 18009.50 | 284.00 | 413.25 | 4108.00 | 126.00 |
| Hunan | 32944.93 | 6726.68 | 22432.75 | 93336.38 | 585.59 | 5519.75 | 19.38 | 4.96 | 3.70 | 40.20 | 671.13 | 84.3 | 1699.38 | 468553.05 | 13744.21 | 5.66 | 7.18 | 48.49 | 1665.13 | | 14149.88 | 262.13 | 151.25 | 2967.63 | 16.00 |
| Guangdong | 86151.59 | 11480.75 | 32014.58 | 513473.75 | 307.90 | 10757.75 | 66.88 | 7.05 | 2.43 | 42.63 | 1700.00 | 95.9 | 2992.63 | 1488786.18 | 45553.34 | 11.96 | 4.56 | 52.11 | 3201.50 | | 34925.00 | 567.88 | 432.38 | 2452.75 | 71.63 |
| Guangxi | 18421.10 | 4867.50 | 19262.23 | 19724.38 | 238.18 | 4168.75 | 24.13 | 7.34 | 2.78 | 39.16 | 881.63 | 86.9 | 1101.38 | 185390.06 | 8073.66 | 3.29 | 6.22 | 58.54 | 835.00 | | 12679.13 | 189.25 | 252.75 | 1543.00 | 2.88 |
| Hainan | 4316.30 | 942.67 | 21819.61 | 2518.75 | 90.96 | 942.75 | 5.25 | 7.77 | 2.36 | 40.55 | 164.63 | 90.3 | 348.38 | 84686.08 | 2396.50 | 1.29 | 6.00 | 56.94 | 393.38 | | 2634.13 | 35.88 | 60.38 | 436.38 | 2.00 |
| Chongqing | 18584.07 | 3082.69 | 23418.48 | 52945.25 | 152.35 | 1617.50 | 9.63 | 3.05 | 3.46 | 40.94 | 331.75 | 92.7 | 791.50 | 330022.22 | 9238.96 | 2.79 | 7.33 | 40.64 | 2133.38 | | 15048.63 | 370.75 | 73.63 | 819.00 | 9.00 |
| Sichuan | 36300.50 | 8257.28 | 20034.35 | 69439.13 | 377.44 | 5711.38 | 10.88 | 2.88 | 3.88 | 39.91 | 1276.00 | 84.2 | 2439.88 | 472988.98 | 17278.72 | 5.93 | 7.11 | 36.27 | 2035.50 | | 38052.00 | 304.38 | 115.63 | 2243.50 | 4.00 |
| Guizhou | 12806.67 | 3650.32 | 16199.87 | 18831.25 | 237.26 | 2346.75 | 7.38 | 6.23 | 3.30 | 38.41 | 223.38 | 81.5 | 1133.75 | 251731.71 | 6039.47 | 4.69 | 7.04 | 39.60 | 487.75 | | 2152.13 | 38.75 | 42.00 | 559.63 | 5.63 |
| Yunnan | 16897.84 | 4731.59 | 17762.80 | 20264.13 | 247.47 | 4031.00 | 13.75 | 5.85 | 3.67 | 37.64 | 636.63 | 89.1 | 1598.38 | 630524.24 | 8072.51 | 3.65 | 6.65 | 51.91 | 827.75 | | 10919.63 | 178.25 | 181.25 | 879.13 | 9.38 |
| Xizang(Tibet) | 1286.93 | 337.88 | 15044.91 | 180.50 | 30.57 | 793.25 | 16.50 | 10.35 | 2.68 | 36.70 | 183.00 | 76.4 | 27.25 | 77228.01 | 668.64 | 0.73 | 5.03 | 12.04 | 34.75 | | 925.13 | 98.13 | 62.75 | 491.13 | 3.25 |
| Shaanxi | 21183.60 | 3853.50 | 20066.56 | 45344.38 | 409.16 | 5734.25 | 39.75 | 3.76 | 3.33 | 40.03 | 843.50 | 87.2 | 1137.38 | 417599.41 | 8975.03 | 5.23 | 6.38 | 42.04 | 995.38 | | 16707.63 | 87.13 | 185.13 | 424.75 | 150.63 |
| Gansu | 7567.06 | 2611.75 | 15531.19 | 10915.38 | 154.47 | 3433.63 | 31.25 | 5.01 | 2.58 | 32.96 | 684.50 | 90.6 | 881.13 | 254912.60 | 3869.76 | 2.65 | 6.59 | 11.30 | 751.25 | | 7972.00 | 255.38 | 117.13 | 611.38 | 46.13 |
| Qinghai | 2606.98 | 588.75 | 18356.21 | 1754.25 | 66.62 | 1159.38 | 12.25 | 7.72 | 2.90 | 32.66 | 250.00 | 84.5 | 468.38 | 66464.67 | 1156.35 | 0.84 | 6.26 | 5.70 | 249.25 | | 1285.25 | 141.13 | 63.00 | 349.00 | 3.38 |
| Ningxia | 3276.91 | 686.13 | 19967.86 | 6453.75 | 31.09 | 987.38 | 7.25 | 8.03 | 3.93 | 39.88 | 170.13 | 87.8 | 330.00 | 94064.68 | 1530.36 | 0.91 | 5.05 | 12.17 | 679.13 | | 4796.38 | 192.25 | 50.88 | 359.50 | 1.63 |
| Xinjiang | 10885.49 | 2429.25 | 19050.48 | 6162.63 | 107.13 | 2691.88 | 13.38 | 8.38 | 2.69 | 38.70 | 591.88 | 82.5 | 502.13 | 223816.27 | 5289.28 | 2.86 | 4.72 | 4.47 | 792.63 | | 12995.00 | 138.00 | 225.75 | 502.50 | 3.88 |
